# Natural products targeting the Nrf2 signaling pathway: potential targets and intervention strategies for the prevention and treatment of colorectal cancer

**DOI:** 10.3389/fphar.2026.1863440

**Published:** 2026-07-17

**Authors:** Meng Jiao, Jing Su, PingPing Li, QianQian Kong, TianYi Feng, Wei Kong

**Affiliations:** 1 The Second Affiliated Hospital of Shandong First Medical University, Ji Nan, China; 2 Taian First People’s Hospital, Taian, China; 3 Affiliated Hospital of Shandong University of Traditional Chinese Medicine, Ji Nan, China

**Keywords:** apoptosis, colorectal cancer, natural products, Nrf2, oxidative stress

## Abstract

Colorectal cancer (CRC) is a highly prevalent gastrointestinal malignancy worldwide, characterised by insidious onset, rapid progression, high tendency for recurrence and metastasis, and resistance to chemotherapy. It poses significant challenges for clinical management and has become a major public health issue threatening human health. Current clinical treatments for CRC remain significantly limited. Traditional chemotherapeutic agents exhibit poor selectivity and pronounced toxic side effects, and long-term use readily induces tumour resistance, resulting in suboptimal therapeutic outcomes. Consequently, the identification of highly effective and safe core therapeutic targets and novel intervention strategies holds significant clinical value. The Nrf2 signalling axis is a core pathway regulating the body’s redox homeostasis, inflammatory responses, and the biological behaviour of tumour cells. Abnormal activation of this pathway can drive the proliferation, invasion, and metastasis of CRC cells, inhibit tumour cell apoptosis, and simultaneously mediate the development of chemotherapy resistance, making it a key molecular target involved in the onset and progression of CRC. Due to their widespread sources, structural diversity, low toxicity, and unique ability to exert synergistic regulation across multiple targets and pathways, natural products represent a vital resource for the development of novel anticancer drugs. A substantial body of research has demonstrated that various bioactive natural products can modulate the mode of cell death in CRC cells, inhibit malignant progression, reverse drug-resistant phenotypes, and improve the tumour immune microenvironment by specifically regulating Nrf2 and the expression of its downstream target genes. This article elucidates the core regulatory mechanisms of the Nrf2 signalling axis in the progression of CRC and summarises the anti-CRC effects and molecular mechanisms of various natural products that target this pathway. It analyses the application advantages and translational bottlenecks of natural products, providing a theoretical basis and new research insights for the development of novel natural anti-CRC drugs and the optimisation of clinical combination therapy regimens.

## 1 Introduction

Colorectal cancer (CRC) is a malignant tumor of the gastrointestinal tract that originates from the epithelial cells of the colorectal mucosa. Its core pathological characteristics include uncontrolled proliferation following malignant transformation of intestinal epithelial cells, dysregulation of apoptosis, and an abnormal increase in invasive and metastatic potential (Li et al., 2021). Without timely and effective intervention, the tumor can progressively advance and metastasize to distant organs, ultimately leading to multi-organ failure and posing a serious threat to patients’ lives and health (Alvarado-Ortiz et al., 2026). Globally, the incidence and mortality rates of CRC have remained persistently high, representing a significant disease burden. It ranks third among all malignant tumors, following breast and lung cancer, accounting for approximately 10% of all new cancer cases worldwide, with a case fatality rate of 9.4%. The persistently high incidence and mortality rates not only place a heavy burden on global healthcare systems but also result in significant socioeconomic losses (Bray et al., 2024).

Although the pathogenesis of CRC has not been fully elucidated, existing research indicates that its development involves complex and interrelated biological processes, including the continuous accumulation of oxidative DNA damage, abnormal activation of oncogenes, inactivation of tumor suppressor genes, the persistent driving role of chronic inflammation, and the remodeling of the tumor immune microenvironment (Lee et al., 2021). Among the various regulatory mechanisms, the abnormal regulation of the nuclear factor erythroid 2-related factor 2 (Nrf2) signaling pathway is a key molecular mechanism mediating the onset and progression of CRC (Abedizadeh et al., 2024). It is important to emphasize that the persistent abnormal activation of the Nrf2 signaling pathway, on the one hand, enhances the antioxidant capacity of tumor cells, clears excess intracellular reactive oxygen species (ROS), reduces oxidative DNA damage, and protects tumor cells from oxidative stress (OS)-induced apoptosis; on the other hand, by regulating the cell cycle, inhibiting ferroptosis, promoting epithelial-mesenchymal transition (EMT), and reshaping the tumor immune microenvironment, it accelerates the proliferation, invasion, and metastasis of CRC cells, while also mediating tumor cell resistance to chemotherapy, radiotherapy, and targeted therapy, thereby becoming a core driving factor in the development of CRC (Abdelgawwad El-Sehrawy et al., 2025).

Current clinical treatment strategies for CRC primarily rely on surgical resection, combined with comprehensive interventions such as chemotherapy, radiotherapy, targeted therapy, and immunotherapy. Commonly used therapeutic agents include 5-FU, oxaliplatin, irinotecan, anti-EGFR monoclonal antibodies, and PD-1/PD-L1 inhibitors. Although these treatments can delay disease progression and prolong patient survival to some extent, they still have significant limitations: low rates of early screening mean that most patients are diagnosed at an advanced stage, missing the optimal window for treatment; chemotherapy drugs lack specificity, and long-term use is often associated with severe adverse effects such as gastrointestinal reactions, bone marrow suppression, and liver and kidney damage (Feng et al., 2021). Therefore, identifying a safe and effective treatment for CRC patients is of paramount importance. Numerous studies have confirmed that the Nrf2 signaling pathway plays a key regulatory role in the pathological progression of CRC. Targeting this pathway can effectively inhibit the proliferation of CRC cells, induce tumor cell apoptosis, reverse treatment resistance, and enhance antitumor immune responses, highlighting its significant value as a potential therapeutic target for the prevention and treatment of CRC (Li W. et al., 2022).

Due to their structural diversity, broad biological activity, low toxicity, and widespread availability, natural products have become a vital source for new drug development and have garnered significant attention in the prevention and treatment of malignant tumors. Many modern anticancer drugs are derived from natural products or their derivatives. Among these, natural products such as flavonoids and terpenoids, medicinal plant extracts, and TCM formulations possess distinct advantages in CRC research due to their inherent safety and the synergistic regulatory effects of their multi-component, multi-target mechanisms; they have been demonstrated to exhibit potent anti-CRC activity. Compared to synthetic drugs targeting a single pathway, these natural products can modulate pathological states through multiple pathways and targets, achieving multifaceted effects such as antioxidant, anti-inflammatory, pro-apoptotic, and anti-invasion and metastasis actions. Recent studies have shown that natural products can exert anti-CRC effects by targeting the Nrf2 signaling axis, regulating its nuclear translocation and the expression of downstream target genes, thereby offering new directions for the prevention and treatment of this disease. This review systematically summarizes the core regulatory mechanisms of the Nrf2 signaling axis in the development of CRC, focuses on elucidating the potential pharmacological pathways through which natural products exert anti-CRC effects via this signaling axis, and explores their application prospects and translational challenges, thereby providing a theoretical basis and innovative ideas for subsequent basic research and clinical translation.

## 2 Methods and literature search strategy

**FIGURE 1:**
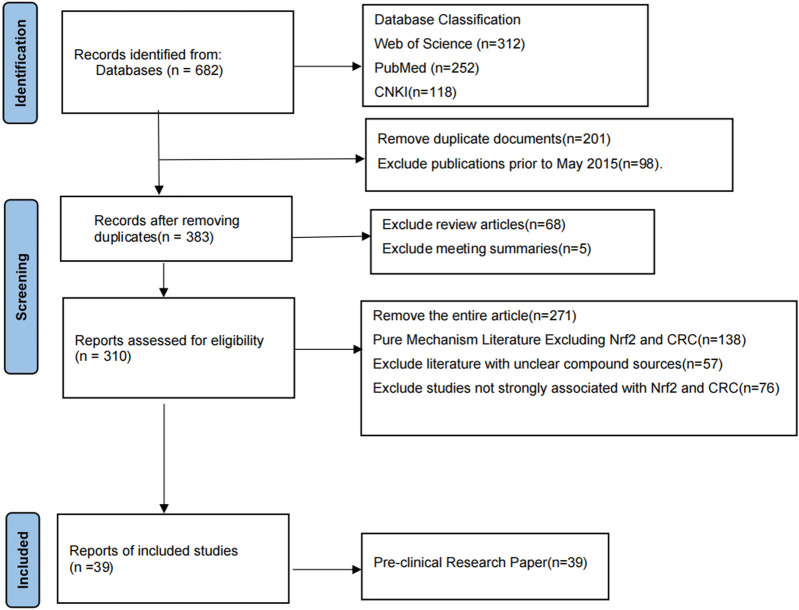
Flowchart of literature search.

This study conducted a systematic literature search in the PubMed, Web of Science, and CNKI databases, with the search period limited to May 2015 to March 2026. To ensure the comprehensiveness and accuracy of the literature retrieval, the search strategy combined Medical Subject Headings (MeSH) terms with free-text keywords, systematically covering core research concepts such as “Nrf2”, “colorectal cancer”, and “natural products/traditional Chinese medicine (TCM) (including monomers, extracts, metabolites, and TCM formulas)”. The initial search identified a total of 682 literature records. These were first screened based on titles and abstracts, followed by an in-depth full-text review of potentially eligible studies. In accordance with the inclusion and exclusion criteria, studies with risks of selection bias, detection bias, reporting bias, or other methodological biases were strictly excluded. Ultimately, 39 high-quality studies meeting the inclusion criteria were selected for in-depth discussion and systematic analysis in this review (Figure 1).

## 3 Overview of the Nrf2 signalling pathway

Nrf2 is a highly evolutionarily conserved basic zinc-finger (bZIP) family transcription factor and a key molecule regulating cellular redox homeostasis (Baird and Yamamoto, 2020). Nrf2 comprises six independent functional domains, Neh1 to Neh6, which work in concert to regulate its own protein stability and transcriptional activity. Among these, the Neh2 domain contains the conserved DLG and ETGE motifs, which enable sustained binding to Kelch-like epoxychloroethane-associated protein 1 (Keap1). Under physiological conditions, this interaction mediates Keap1-dependent ubiquitination and proteasomal degradation of Nrf2, maintaining low basal levels of Nrf2 within the cell and preventing abnormal activation of the pathway. Conversely, the Neh1 domain mediates the formation of a heterodimer between Nrf2 and small Maf proteins. This complex specifically recognises the antioxidant response element (ARE) within the promoters of downstream target genes, thereby initiating the transcription of antioxidant and detoxification-related genes (Bellezza et al., 2018). Keap1, as the primary negative regulator of Nrf2, functions through its three core domains: BTB, IVR and Kelch. The BTB domain promotes Keap1 homodimerisation whilst recruiting the Cul3-Rbx1 E3 ubiquitin ligase complex, providing a structural scaffold for the ubiquitination of Nrf2 (Suzuki et al., 2025). The IVR region is rich in redox-sensitive cysteine residues and serves as a key site for sensing OS and electrophilic stimuli, triggering conformational changes in Keap1. The C-terminal Kelch domain forms a characteristic β-propeller structure, which specifically binds to and anchors the Nrf2 protein (Wu J. et al., 2023). These three domains collectively constitute the classic Keap1-dependent pathway, precisely regulating the homeostatic degradation and functional balance of Nrf2.

In addition to the classical Keap1-dependent pathway, Nrf2 activity is finely regulated by various post-translational modifications and non-Keap1-dependent regulatory factors, forming a complex, multi-tiered signalling regulatory network. Ubiquitination, phosphorylation and acetylation are the primary post-translational modifications regulating Nrf2 function, and are involved throughout the regulation of Nrf2 stability, subcellular localisation and transcriptional activity (Yang et al., 2025). Ubiquitination is the key modification determining the half-life of the Nrf2 protein and can regulate Nrf2 degradation via both Keap1-dependent and Keap1-independent pathways; phosphorylation exerts a bidirectional regulatory effect on Nrf2, with different upstream kinases targeting distinct sites on Nrf2 to either promote its degradation or enhance its stability and nuclear translocation capacity (Komeda et al., 2026). Acetylation occurs primarily on nuclear Nrf2, where it reshapes the protein’s spatial conformation to enhance its binding affinity for ARE elements, thereby amplifying transcriptional activation. Complex cross-talk exists between various post-translational modifications, enabling dynamic fine-tuning of signalling (Xu et al., 2021). Furthermore, factors such as β-TrCP, HRD1 and WDR23 can regulate Nrf2 homeostasis independently of Keap1: β-TrCP and HRD1, which is localised to the endoplasmic reticulum, can mediate the ubiquitination and degradation of Nrf2, thereby inhibiting its abnormal accumulation; the scaffold protein WDR23 can retain Nrf2 in the cytoplasm, blocking its nuclear translocation and transcriptional activation. Multiple regulatory mechanisms complement and synergise with one another to jointly maintain the functional stability of Nrf2 under physiological conditions (Hayes et al., 2025).

Under normal physiological conditions, Nrf2 acts as a key stress sensor in cells, responding to external stimuli such as OS and electrophilic damage. Stress signals can induce a conformational change in Keap1, prompting Nrf2 to dissociate from the Keap1 complex and translocate to the nucleus, converting cytoplasmic stress signals into nuclear transcriptional signals and initiating cellular protective response programmes (O’Rourke et al., 2024). The Nrf2 pathway is extensively involved in biological processes such as antioxidant defence, detoxification of exogenous toxins, inflammation regulation, and cell fate determination, exerting bidirectional regulatory effects on cell proliferation, apoptosis, autophagy, and metabolic reprogramming (Zhang et al., 2025). Upon nuclear translocation, Nrf2 significantly upregulates the expression of antioxidant genes such as superoxide dismutase (SOD), glutathione peroxidase (GPx) and haem oxygenase-1 (HO-1), effectively scavenging excess ROS (ROS) within the cell, neutralising toxic electrophiles, balancing the secretion of pro-inflammatory and anti-inflammatory factors, and mitigating chronic inflammatory damage. It is thus evident that Nrf2 serves as a central molecular hub linking upstream stress signals to downstream cellular defence responses (Kasai et al., 2020). Under normal conditions, the Nrf2 pathway can be rapidly activated and promptly terminated, maintaining cellular redox balance; however, persistent pathological stimuli disrupt this homeostatic network, thereby driving the onset and progression of tumours.

**FIGURE 2:**
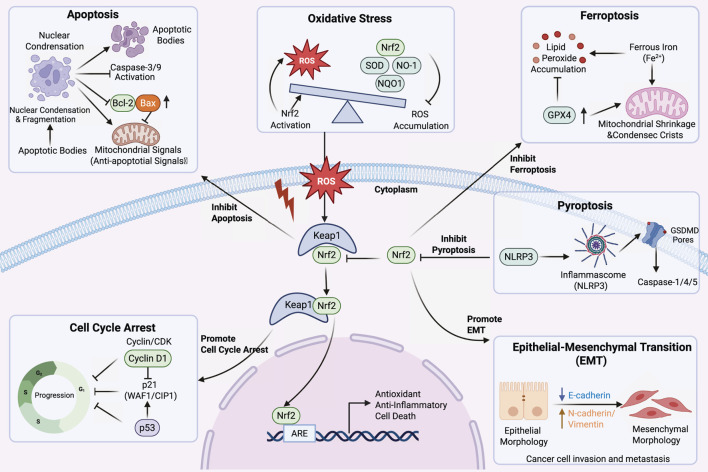
Schematic diagram of Nrf2 involvement in CRC mechanisms. Figure 2 was created using BioRender.

Extensive research has confirmed that the persistent abnormal activation of Nrf2 is closely associated with the development, progression and metastasis of CRC (Li C. X. et al., 2022). In colorectal tissues, which are chronically exposed to an environment of chronic inflammation and persistent oxidative damage, the classical Keap1-mediated degradation pathway of Nrf2 is inhibited, whilst non-Keap1-dependent regulatory pathways are disrupted. This ultimately leads to the massive accumulation of Nrf2 in the cytoplasm and its continuous nuclear translocation, thereby activating downstream transcriptional programmes. Persistently activated Nrf2 can reshape the malignant phenotype of CRC cells in multiple ways: by upregulating antioxidant enzymes to scavenge excess ROS, remodeling the tumour oxidative microenvironment, creating favourable conditions for tumour cell survival (Acevedo-León et al., 2022). By upregulating the expression of GPX4 and SLC7A11, it inhibits LPO and ferroptosis, whilst simultaneously attenuating pyroptosis-mediated inflammatory clearance and enhancing cellular anti-apoptotic capacity, thereby supporting tumour cell survival through multiple pathways (Wang M. et al., 2024). By regulating cell cycle-related proteins to drive abnormal proliferation of tumour cells (Nie et al., 2026). By activating EMT-related transcriptional pathways and upregulating EMT marker molecules, significantly enhancing the migratory and invasive capabilities of tumour cells, thereby accelerating the malignant progression and distant metastasis of CRC (Zhang et al., 2026). In summary, the dysregulation of the Nrf2 regulatory network is a key driver of malignant progression in CRC. Targeted inhibition of abnormal Nrf2 activation can effectively reverse various malignant phenotypes of the tumour and restore normal cellular physiological functions, thereby providing novel targets and directions for translational research in the clinical prevention and treatment of CRC, with significant clinical application value (Figure 2).

## 4 Mechanistic role of Nrf2 in colorectal cancer

### 4.1 Inducing ferroptosis in cells

Ferroptosis is a process distinct from apoptosis, autophagy and necrosis. Its core characteristics include an imbalance in intracellular iron metabolism, the massive accumulation of LPO products and the failure of the antioxidant system; it is a key cellular event regulating tumourigenesis and progression (Dixon and Olzmann, 2024). Research by Sun et al. has confirmed that the occurrence of ferroptosis primarily relies on the Fe^2+^-mediated Fenton reaction, which catalyses the LPO of polyunsaturated fatty acids on the cell membrane. This is accompanied by the depletion of glutathione (GSH) and significant decline in glutathione peroxidase 4 (GPX4) activity, ultimately leading to the destruction of cellular structures and cell death (Sun et al., 2020). Furthermore, ferroptosis is characterised by typical morphological features such as mitochondrial volume reduction, increased membrane density and decreased cristae structure. Its onset and progression are closely linked to the precise regulation of iron metabolism, lipid metabolism and the antioxidant system, and it has now become an important area of research in targeted cancer therapy (Zhou et al., 2024).

As a key antioxidant transcription factor in the body, Nrf2 has attracted widespread attention in CRC treatment research and represents a potentially valuable therapeutic target. Studies by An et al. have shown that inhibition of Nrf2 promotes LPO, upregulates intracellular Fe^2+^ accumulation and the massive production of ROS, induces ferroptosis, and suppresses the viability and clonogenic potential of rectal cancer cells, thereby exerting an antitumour effect (An et al., 2026). The combination of Nrf2 inhibitors with chemotherapeutic agents such as oxaliplatin can significantly enhance the efficacy of chemotherapy and reverse tumour resistance by increasing sensitivity to ferroptosis (Bai et al., 2025). Furthermore, research by Wei et al. indicates that downregulating Nrf2-mediated antioxidant defence weakens cellular resistance to ferroptosis and enhances sensitivity to cetuximab, thereby exerting an anti-tumour effect (Wei et al., 2023).

The above studies indicate that targeting the bidirectional regulation of the Nrf2-mediated ferroptosis pathway offers a novel approach for precision intervention in CRC. This holds promise for driving a shift in CRC treatment from “palliative care” to “precision targeting”, providing crucial theoretical support for enhancing clinical efficacy and improving prognosis.

### 4.2 Inhibition of the inflammatory response

The inflammatory response is an active immune defence process by which the body responds to injury, infection and stress stimuli. Characterised by immune cell infiltration, the release of inflammatory mediators and disruption of the tissue microenvironment, it is a key pathological process driving tumour initiation, progression and metastasis. Under physiological conditions, a moderate inflammatory response can eliminate abnormal cells, repair damaged tissues, and maintain the homeostasis of the intestinal environment (Tong et al., 2024). However, persistent abnormal activation of inflammation leads to a chronic, low-grade inflammatory microenvironment in the intestine, which continuously damages normal tissues, induces cellular genetic mutations, and promotes malignant lesions. Chronic intestinal inflammation is a central driver of the development of CRC. It activates both classical pro-inflammatory pathways and non-classical pathways that rely on inflammasome assembly, synergistically amplifying inflammatory cascades, disrupting the intestinal mucosal barrier, and remodelling the tumour microenvironment, thereby providing favourable pathological conditions for the proliferation, invasion, and metastasis of CRC cells (Guo et al., 2021).

As a key transcription factor at the core of the body’s anti-inflammatory and antioxidant defences, Nrf2 has emerged as a promising potential target for the treatment of CRC through its precise regulation of the intestinal inflammatory response. Within the pathological microenvironment of CRC, mucosal damage and excessive OS continuously activate inflammatory pathways, leading to the uncontrolled activation of the inflammatory response (Bardelčíková et al., 2023). On the one hand, persistent chronic inflammation repeatedly damages normal intestinal mucosal epithelial cells, inducing abnormal cell proliferation and genetic mutations, thereby driving the transformation of colorectal adenomas into malignant tumours (Schmitt and Greten, 2021). On the other hand, an overactivated inflammatory response can reshape the tumour immune microenvironment by secreting large amounts of pro-inflammatory cytokines, thereby suppressing the body’s anti-tumour immune response and facilitating cancer cell immune evasion, proliferation, invasion and distant metastasis (Duan et al., 2025).

The above studies indicate that uncontrolled chronic inflammation is a central driver of CRC progression. Nrf2 negatively regulates intestinal inflammatory responses via multiple pathways and inhibits the remodelling of the inflammatory microenvironment; the anti-inflammatory effects it mediates play a key anti-tumour role. Targeting Nrf2 to inhibit abnormal intestinal inflammatory responses offers a new perspective on elucidating the pathological mechanisms by which chronic inflammation mediates the malignant transformation of CRC. It also lays an important theoretical foundation for the development of precision targeted therapies for CRC and intervention strategies for inflammation-associated tumours, possessing significant value for basic research and clinical translation.

### 4.3 Regulation of oxidative stress

The disruption of OS homeostasis is a core pathological mechanism driving the initiation, proliferation and malignant progression of CRC, characterised by the abnormal accumulation of ROS, nitric oxide (NO) and other oxidatively active mediators within cells, thereby disrupting the dynamic equilibrium between the body’s oxidative and antioxidant systems (Jannuzzi et al., 2022). Under physiological conditions, the body’s endogenous antioxidant system can promptly clear baseline levels of ROS, maintaining the structural and functional stability of intestinal epithelial cells. However, the persistent low-grade chronic OS within the CRC microenvironment acts as a key oncogenic signal, continuously activating pathways associated with tumour proliferation, anti-apoptosis, and invasion and metastasis. This induces the formation of a tumour hypoxic microenvironment and a chemoresistant phenotype, ultimately accelerating the malignant progression of CRC (Cao J. F. et al. ,2025).

The Keap1-Nrf2 pathway is a core signalling pathway that mediates intestinal OS homeostasis and regulates the pathological progression of CRC, comprising a complete regulatory axis consisting of the upstream regulator Keap1 and downstream effector molecules such as NQO1 and HO-1. Under physiological conditions, cytoplasmic Keap1 mediates the ubiquitination and degradation of Nrf2, thereby inhibiting abnormal activation of the pathway, preventing excessive synthesis of ROS, and maintaining intestinal tissue homeostasis (Tripathi et al., 2025). A small amount of Nrf2 that stably translocates into the nucleus binds to antioxidant response elements, initiating the basal transcriptional expression of downstream NQO1 and HO-1. NQO1 catalyses the reduction and detoxification of quinone-based toxic substances, thereby blocking OS cascade reactions; HO-1 generates antioxidant active substances through the degradation of haem, scavenging excess intracellular ROS and NO. Together, they maintain the redox balance in normal intestinal cells and exert a tissue-protective effect (Wang et al., 2019).

In the pathological context of CRC, tumour cells can abnormally hijack the Keap1-Nrf2 pathway by upregulating Keap1 and Nrf2 expression to sustain activation of the pathway and inducing abnormally high expression of downstream NQO1 and HO-1 (Zhang et al., 2019). Overactivated downstream effector molecules can establish a potent, tumour-specific antioxidant system, effectively clearing oxidative damage signals within tumour cells, antagonising apoptosis and maintaining tumour cell survival. Concurrently, the abnormal accumulation of HO-1 can reshape the tumour metabolic microenvironment and promote angiogenesis, further facilitating tumour proliferation, invasion and malignant progression. Targeting Nrf2 can precisely block the pathological activation of this pathway, exerting a bidirectional antioxidant and anti-tumour effect through coordinated regulation of upstream and downstream components (Wang et al., 2019). In normal intestinal tissue, targeting Nrf2 can inhibit the abnormal accumulation of oxidative mediators, moderately regulate the basal expression of NAD(P)H: Quinone Oxidoreductase 1(NQO1) and HO-1, alleviate chronic oxidative damage, and eliminate the pathological basis for tumour development. In CRC cells, however, this intervention accelerates Nrf2 protein degradation, blocks its nuclear transcription process, significantly downregulates the pathological overexpression of NQO1 and HO-1, and completely dismantles the tumour cells’ antioxidant defence system (Tsai et al., 2022). The loss of antioxidant defence in tumour cells leads to massive accumulation of ROS and NO, inducing DNA damage and Lipid Peroxidation (LPO), thereby activating endogenous apoptotic pathways and efficiently eliminating tumour lesions. Concurrently, Nrf2-targeted intervention reverses hypoxia-mediated chemoresistance, significantly enhancing the drug sensitivity of tumour cells.

The above studies indicate that targeting Nrf2 can differentially regulate OS levels in normal and tumour tissues via the complete Keap1-Nrf2-NQO1/HO-1 regulatory axis, thereby achieving precise therapeutic effects in inhibiting CRC proliferation, reversing drug resistance, and suppressing malignant progression.

### 4.4 Regulation of apoptosis

Apoptosis is a controlled, programmed cell death process in the body that enables the timely clearance of mutated or damaged abnormal cells, thereby maintaining the physiological homeostasis of intestinal tissues. Dysregulation of the apoptotic pathway constitutes a key pathological basis for the development, proliferation and invasion of CRC (Fan et al., 2021). CRC cells use various mechanisms to suppress their own apoptosis, evading the body’s immune and metabolic surveillance, thereby achieving malignant, uncontrolled proliferation. Nrf2 is a key antioxidant transcription factor that participates in the development and progression of CRC by bidirectionally regulating the apoptosis pathway. It is an important therapeutic target for the treatment of this disease. By precisely regulating the expression and activity of Nrf2, it is possible to effectively intervene in the tumour apoptosis process and inhibit the proliferation and progression of CRC (Jin et al., 2025).

Nrf2 can regulate apoptosis through inhibitory mechanisms, thereby hindering the progression of CRC. Within the tumour microenvironment, Nrf2 is abnormally overexpressed in CRC cells, continuously activating downstream anti-apoptotic and antioxidant-related genes. This reduces the accumulation of ROS within the cells, helping tumour cells resist oxidative damage and evade programmed apoptosis, thereby creating conditions conducive to tumour proliferation and invasion (Li J. et al., 2022). Studies have shown that following specific inhibition of Nrf2, the antioxidant defence system of CRC cells is disrupted, leading to a massive accumulation of ROS. This damages the structure and function of tumour cell mitochondria, effectively activates endogenous apoptotic pathways, and significantly promotes apoptosis in CRC cells. This mechanism can directly curb the unlimited proliferation of tumour cells, block tumour tissue growth, invasion and metastasis, and inhibit the malignant progression of CRC at the lesion level (Li J. et al., 2022).

Moderate activation and promotion of Nrf2 expression can similarly regulate the apoptotic process, ultimately inhibiting the proliferation and progression of CRC, complementing the targets and mechanisms of Nrf2 inhibition (Mizukami et al., 2024). In the early stages of CRC, the intestinal mucosa is subjected to prolonged inflammatory stimulation and OS damage. Normal intestinal epithelial cells are prone to DNA damage and abnormal apoptosis, which subsequently trigger mucosal hyperplasia and malignant transformation, gradually leading to the formation of tumour lesions. At this stage, moderate promotion of Nrf2 activation can significantly enhance the antioxidant and anti-apoptotic capabilities of normal intestinal cells, reducing damage and abnormal apoptosis in these cells, thereby preventing the malignant transformation of the intestinal mucosa and blocking the initiation of tumour development at its source (Hamamah et al., 2024). This protective effect on normal cells and its ability to inhibit abnormal carcinogenesis can effectively prevent the progression of precancerous lesions to malignant tumours, thereby indirectly suppressing the proliferation and progression of CRC, and providing an important avenue for the early prevention and control of the disease.

In summary, as a central molecule in the regulation of apoptosis, Nrf2 plays a key role in the pathological progression of CRC by participating in the fine-tuning of apoptosis through multiple mechanisms. By targeting Nrf2 and modulating its regulatory role in apoptosis, it is possible to effectively restore the apoptotic capacity of CRC cells, thereby inhibiting tumour proliferation and drug resistance, and providing new insights and potential targets for clinical intervention in CRC.

### 4.5 Induction of cell cycle arrest

Cell cycle arrest is a crucial defense mechanism by which cells respond to external stimuli (such as DNA damage and OS). It is essential for maintaining genomic stability and suppressing abnormal proliferation, and plays a central regulatory role in the pathological progression of tumorigenesis. The orderly progression of the cell cycle is precisely regulated by multiple molecules, including cyclin-CDK complexes and tumor suppressor genes. When cells are damaged, they initiate a cell cycle arrest program, causing the cell to stall at specific phases such as G1 or G2/M, thereby providing time for DNA repair; if the damage cannot be repaired, apoptosis is induced (Sun et al., 2022).

By regulating cell cycle arrest, Nrf2 has emerged as a key potential therapeutic target for CRC. Its dysregulation is closely associated with the onset, progression, and chemoresistance of the disease (Hu et al., 2024). Nrf2 is frequently overexpressed in CRC tissues. By inhibiting cell cycle arrest, it promotes uncontrolled proliferation of tumor cells while enhancing their DNA damage repair capacity, leading to chemoresistance (Sadeghi et al., 2017). Notably, in an oxaliplatin-induced CRC model, the Nrf2 inhibitor ML385 restores G2/M phase arrest in tumor cells by inhibiting Nrf2 activity, thereby enhancing the cytotoxic effect of the chemotherapeutic agent and downregulating the expression of proliferation markers such as Ki67 (Wang C. et al., 2018).

These studies demonstrate that Nrf2 plays a crucial role in regulating cell cycle arrest, particularly in establishing key pathological mechanisms underlying the onset and progression of CRC. By targeting Nrf2 and its associated cell cycle regulatory factors, it is possible to effectively induce cell cycle arrest in CRC cells, inhibit tumor proliferation, and reverse chemotherapy resistance. Specific Nrf2 inhibitors or natural bioactive compounds, by regulating Nrf2-mediated cell cycle arrest, offer a novel intervention strategy for CRC and provide a highly promising targeted direction for its clinical treatment.

### 4.6 Inhibition of epithelial-mesenchymal transition

EMT is a dynamic process in which epithelial cells, under specific physiological or pathological conditions, lose their epithelial phenotype and acquire a mesenchymal phenotype through cellular reprogramming. It is a key driving mechanism in the initiation, progression, invasion, and metastasis of CRC (Shin et al., 2023). Under normal physiological conditions, EMT participates in physiological processes such as embryonic development and tissue repair, maintaining tissue homeostasis. In pathological conditions, however, abnormal activation of EMT leads to loss of epithelial cell polarity and disruption of intercellular junctions. This is accompanied by downregulation of epithelial markers (such as E-cadherin) and upregulation of mesenchymal markers (such as vimentin and the Snail transcription factor), endowing cells with enhanced migration and invasion capabilities as well as anti-apoptotic properties, while also contributing to the development of tumor drug resistance (Zhang et al., 2021). During the pathological progression of CRC, tumor tissues are continuously subjected to a multifaceted pathological state characterized by chronic inflammatory infiltration, OS imbalance, and the massive release of pro-metastatic factors. This leads to abnormal activation of EMT, promoting tumor cell invasion and metastasis. Consequently, by regulating the EMT process, Nrf2 has emerged as an important potential therapeutic target for CRC (Schiavoni et al., 2025).

The above studies indicate that Nrf2 plays a crucial role in regulating the dynamic balance of EMT. By targeting Nrf2, it is possible to effectively inhibit the abnormal activation of EMT in CRC cells, reduce tumor invasion and metastasis, and thereby slow the progression of CRC. Therefore, targeting Nrf2 to regulate the EMT process offers a novel intervention strategy for CRC and provides a potentially effective therapeutic target for its clinical treatment.

## 5 Natural medicines targeting the Nrf2 signalling pathway to intervene in the progression of colorectal cancer

**FIGURE 3:**
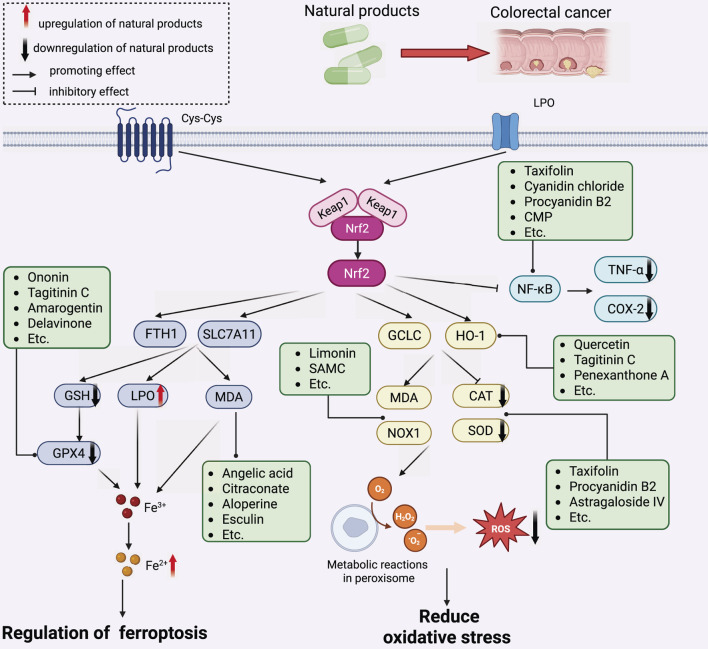
Mechanisms by which natural products target Nrf2 to treat CRC through regulating ferroptosis and reducing oxidative stress. Figure 3 was created using BioRender.

**FIGURE 4:**
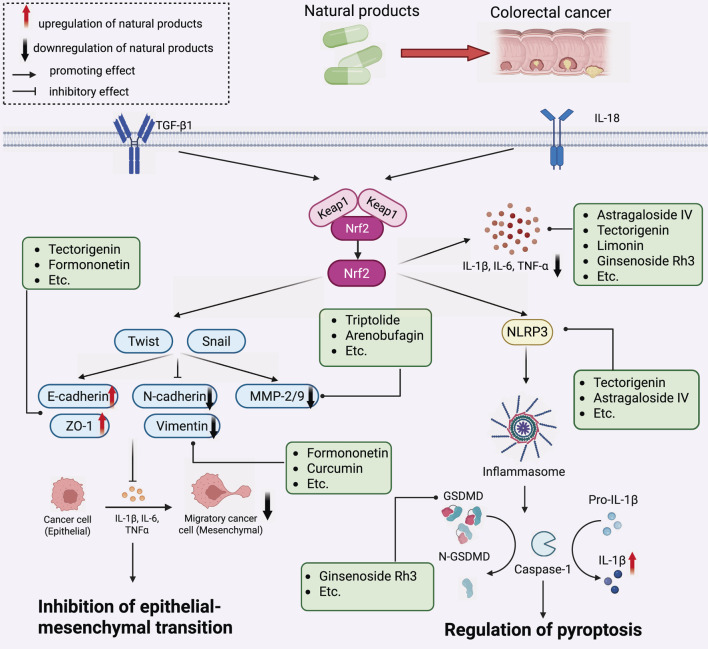
Mechanisms by which natural products target Nrf2 to treat CRC through inhibiting Epithelial-Mesenchymal Transition and regulating pyroptosis. Figure 4 was created using BioRender.

**FIGURE 5:**
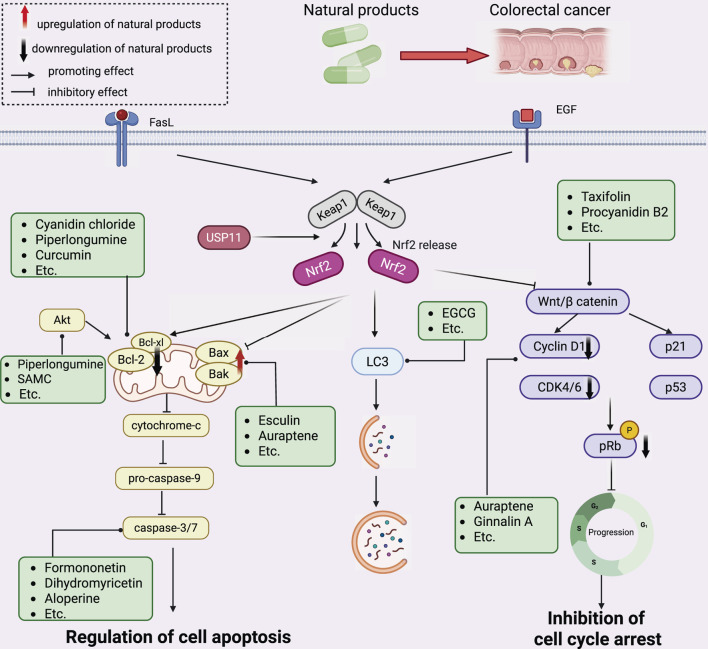
Natural products target Nrf2 to treat CRC: regulation of cell apoptosis and induction of cell cycle arrest.

Numerous studies have confirmed that various natural medicines, such as flavonoids, terpenoids, Chinese herbal extracts, and TCM formulations, can target Nrf2. By regulating the activation status of this pathway and the expression patterns of its downstream target genes, they restore the redox homeostasis of colorectal tumor tissues, thereby intervening in the malignant progression of the tumor. Under this regulatory effect, the excessive accumulation of ROS within tumor cells is effectively cleared, malignant proliferation is significantly reduced, apoptosis is efficiently induced, and the expression of molecules associated with invasion and metastasis is suppressed. Concurrently, tumor-associated inflammatory responses are alleviated, and the pro-tumor properties of the tumor microenvironment are reversed. These natural products may thus represent highly promising candidate drugs for targeted intervention in CRC (Tables 1, 2; Figures 3–5).

**TABLE 1:** Natural drugs regulate Nrf2 signaling pathway to treat CRC.

Extract	*Origination*	Structure	Dosage	Model	Biological effects	Results	Limitations	References
Ononin	*Astragalus mongholicus Bunge*	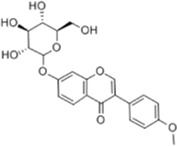	60 mg/kg	HCT116/MC38 cells	ROS↑MDA↑GSH↓GPX4↓	Inducing ferroptosis in CRC cells and enhancing the efficacy of immune checkpoint inhibitor therapy	There is poor oral bioavailability, and biomarkers related to clinical translation have not yet been identified.	Gui et al. (2025)
			2–16 μM	MC38 tumor-bearing mice				
Tectorigenin	*Iris domestica (L.) Goldblatt and Mabb.*	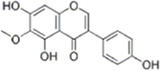	40,80 mg/kg	CT26 tumor-bearing mice	IL-1β↓IL-6↓TNF-α↓IL-10↑ZO-1↑Occludin↑E-Cadherin↑	Inhibits the growth of CT26 transplanted tumors, promotes tumor cell apoptosis, and inhibits proliferation	The differential regulatory effects of Nrf2 in tumor and normal tissues have not been clearly established.	Ma et al. (2025)
			50,100 μmol/L	Caco-2 colon cancer cells				
Quercetin	*Styphnolobium japonicum (L.) Schott*	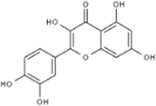	10,40 μM	HCT116 cells	SOD↓CAT↓GPx↓GR↓	Inhibits the proliferation of colon cancer cells and drug-resistant cells, and induces apoptosis	The study relied solely on a single colorectal cancer cell line, lacking supporting evidence from multiple cell lines and primary cells.	Tang et al. (2023)
Taxifolin	*Pseudotsuga menziesii (Mirb.) Franco*	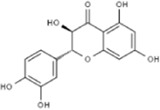	30 mg/kg	DMH-induced mice colon cancer model	CEA↓LDH↓TNF-α↓COX-2↓Nrf2↑	Reduce inflammatory responses in the tumor microenvironment and inhibit abnormal proliferation of colonic epithelial cells	Limited to animal models; lacks data on pharmacokinetics, dose-response relationships, long-term safety, and clinical translation	Manigandan et al. (2015)
Cyanidin chloride	*Vaccinium corymbosum L.*	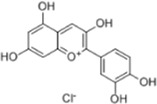	10–100 μM	HCT116,HT29,SW620 cells	Bax↑Cleaved caspase-3↑PARP↑XIAP↓Bcl-2↓	Inhibition of colon cancer cell proliferation	Lack of *in vivo* animal testing	Lee et al. (2020)
Formononetin	*Astragalus mongholicus Bunge*	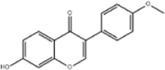	20 mg/kg	Establishment of a BALB/c mouse colorectal cancer model using CT26 cells	Claudin-1↑Occludin↑ZO-1↑Cleaved-caspase 3↓ROS↓NO↓	Enhances small intestinal absorption in mice and improves pathological damage to intestinal tissue	No normal mouse radiotherapy control group was established; the multi-target synergistic mechanism and clinical translation potential still require further validation.	Tang et al. (2025)
Dihydromyricetin	*Vitis vinifera L.*	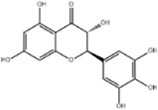	25,50,100 μmol/L	HCT116 cells	CDCF↑MRP2↓Nrf2↓	Reversing multidrug resistance and enhancing the antitumor effects of oxaliplatin	There is a lack of data on efficacy and safety in animals, and other regulatory targets beyond the Nrf2 pathway have not been identified.	Wang et al. (2017)
Tagitinin C	*Tithonia diversifolia (Hemsl.) A.Gray*.	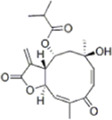	5,10,20 μM	SW480,DLD1,HCT116 cells	ROS↑GSH↓HO-1↑	Inhibits the proliferation and migration of colorectal cancer cells and induces cell cycle arrest	The safety profile, targeted delivery efficiency, and duration of action have not yet been established.	Wei et al. (2021)
Triptolide	*Tripterygium wilfordii Hook.f.*	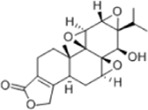	0,25,50,100 nmol/L	HT29 cells	MMP-2↓MMP-9↓Nrf2↓	Inhibiting the proliferation and invasion of colorectal cancer	No *in vivo* animal studies have been conducted, and the agonistic or inhibitory effects on molecules upstream and downstream of this pathway have not been validated.	Wang M. et al. (2024)
Astragaloside IV	*Astragalus mongholicus Bunge*	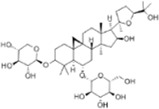	20,40,80 mg/kg	AOM/DSS-induced mice	IL-1β↓IL-6↓TNF-α↓SOD↑CAT↑GSH↑	It can significantly reduce pathological damage to colonic tissue, improve colonic shortening, and reduce the number of tumors	The regulatory role of astragaloside IV in the polarization of intestinal immune cells has not been thoroughly investigated	Liang et al. (2023)
			5,20,50 μM	IEC-6 cells				
Limonin	*Citrus aurantium f. Aurantium*	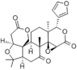	50 mg/kg	Mice with combined AOM/DSS induction	MDA↓PGE2↓TNF-α↓Nrf2↑SOD2↑GSH↑	Strengthens the body’s antioxidant defense system, reduces oxidative stress damage and chronic inflammatory responses, and protects the gut	No long-term toxicity studies, metabolic stability analyses, or clinical trials in humans have been conducted.	Ishak et al. (2021)
Ginsenoside Rh3	*Panax ginseng C.A.Mey.*	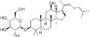	10–160 μM	HT29,HCT116 cells	IL-1β↓IL-18↓SLC7A11↓ROS↑MDA↑	Inhibits tumor cell proliferation, reduces clonogenicity, and significantly decreases the volume and weight of transplanted tumors	Validated only at the cellular and nude mouse levels; lacks evidence from large animal studies and clinical translation	Wu Y. et al. (2023)
			20 mg/kg	Naked mice xenograft model				
Amarogentin	*Centaurium erythraea Rafn*	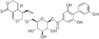	20–160 μg/mL	HCT116,HT-29 cells	ROS↑MDA↑Fe^2+^↑Ki-67↓	Inhibiting cell migration, invasion, and the EMT process by inducing ferroptosis	No clinical validation or drug interaction studies have been conducted	Wang et al. (2025)
			0.2 μg/g	Naked mice subcutaneous tumor xenograft model				
Delavinone	*Fritillaria delavayi Franch*	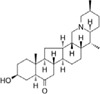	200 μM	HT29,HCT116 cells	GPX4↓SLC7A11↓Ki67↓	Improved weight loss, colon shortening, and tumor burden in mice, and reduced pathological damage to the colon.	This study did not systematically evaluate the safety of long-term administration, pharmacokinetic characteristics, or clinical translation potential.	Zhang et al. (2025)
			2.5.10 mg/kg	AOM/DSS model mice				
Piperlongumine	*Piper longum L.*	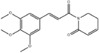	5 μM	HCT-8/5-FU cells	P-gp↓Bcl-xL↓TrxR↓p-Akt↓	Significantly inhibits tumor growth	Not covered by other colorectal cancer drug resistance models	Zhou et al. (2025)
			2.5 mg/kg	Naked mice xenograft				
Cepharanthine	*Stephania japonica (Thunb.) Miers*	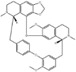	5,10,20 μM	SW480,LOVO,HCT116,SW620 cells	TfR-1↑ACSL4↑GPX4↓CAT↓SOD↓TfR-1↑ACSL4↑	Significantly inhibits tumor growth	The study was conducted solely using *in vitro* cell experiments and lacks validation in animal models.	Sharma et al. (2024)
Aloperine	*Lablab purpureus (L.) Sweet*	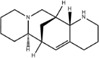	200,300 μM	HCT116,SW480 cells	Bax↑Caspase-3↑Bcl-2↓GSH↓Fe^2+^↑	Inhibit cell proliferation	There is a lack of *in vivo* animal models to validate antitumor efficacy, dosage, and safety.	An et al. (2026)
Quinacrine	*-*	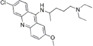	0–5 μM	HCT116,HT29 cells	Nrf2↑	Attenuate tumor drug resistance phenotypes	Does not address human pharmacokinetics, long-term safety, or monitoring for drug-resistant mutations	Kim et al. (2019)
			100 mg/kg	Xenograft nude mouse model				
Curcumin	*Curcuma longa L.*	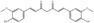	15 μmol/L	HCT116,RKO,SW48 cells	KEAP1/Nrf2↑	Inhibits cell migration and invasion	Clinical translation remains limited by low *in vivo* bioavailability and insufficient targeting and accumulation efficiency	Liu et al. (2023)
			15 μmol/L	Tail vein injection model in NOD/SCID mice				
Epigallocatechin gallate	*Camellia sinensis (L.) Kuntze*	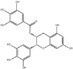	12.5 μmol/L	HCT116 cells	LC3↑Caspase-9↑	Significantly inhibits cell proliferation and reduces the clonogenic rate	No *in vivo* animal studies or clinical trials have been conducted.	Enkhbat et al. (2018)
Hydroxytyrosol	*Olea europaea L.*	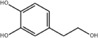	50–200 µM	HCT116 cells	SLC7A11↓GPX4↓Nrf2↓NQO1↓Tfr1↑	Inhibits colony formation and cell migration, and causes damage to the cytoskeletal structure	No evaluation of efficacy and safety in animals has been conducted	Li et al. (2025)
			40–320 µM	SW480 cells				
Ginnalin A	*Acer tataricum subsp. Ginnala (Maxim.) Wesm.*	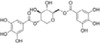	20,40,80 μM	HCT116,SW480,SW620 cells	Nrf2↑HO-1↑NQO1↑Keap1↓p62↑	Inhibit the proliferation of tumor cells	Lacks validation in animal models	Bi et al. (2018)
Procyanidin B2	*Vitis vinifera L.*	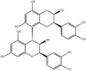	30,50,100 mg/kg	DSS-induced colitis and colitis-associated cancer models	Nrf2/ARE↑NF-κB p65↓	Reduce the incidence and grade of CRC	Issues such as pharmacokinetics and safety require further study	Zhu et al. (2021)
			20 μM	NCM460 cells				
Esculin	*Fraxinus chinensis subsp. Rhynchophylla (Hance) A.E.Murray*	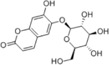	20,40,80 μM	HCT116 cells	COX2↑ACSL4↑FTH1↓ROS↑MDA↑Fe^2+^↑	Alleviated weight loss in mice, reduced the number of colon tumors, and improved histopathological damage in colon tissue	The safety of the drug in normal intestinal cells has not been systematically evaluated, and its *in vivo* pharmacokinetics, bioavailability, and metabolic pathways remain unclear.	Ji et al. (2024)
			20,40 mg/kg	AOM/DSS-induced model mice				
Auraptene	*Citrus aurantium f. Aurantium*	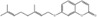	50,100,200 μM	CT26 cells	Bcl-2↓Nrf2↓Cyclin D1↓CAT↓GSH↓FRAP↓	Induce apoptosis in tumor cells and cause cell cycle arrest	No studies have been conducted on the combination of this drug with 5-FU or on its pharmacokinetics, and there is a lack of long-term safety data.	Ebrahimi et al. (2023)
			50,100,200 μM	Balb/C mice CRC xenograft model				
Angelic acid	*Angelica sinensis (Oliv.) Diels*	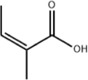	200 μM	DLD1,SW480 cells	MDA↑CHAC1↑PTGS2↑	Significantly inhibits tumor growth and shows no significant organ toxicity in mice	Lack of target validation beyond molecular docking; no comparative studies on combination therapy with first-line chemotherapy drugs	Cao J. F. et al. (2025)
			8 mg/kg	CT26 cell allogeneic transplantation mice model				
Arenobufagin	*Bufo gargarizans*	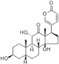	20–80 nM	HCT116,SW620 cells	c-MYC/MAX↓Nrf2↓	Inhibiting cell migration, invasion, and lung metastasis	No pharmacokinetic or long-term safety evaluations have been conducted	Wang Y. et al. (2024)
			0.5,1,2 mg/kg	NOD/SCID mouse lung metastasis model				
Brassinin	*Brassica rapa L.*	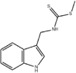	1,50,100,200,400 μM	RKO,HCT116 cells	p62/NRF2/HO-1↓	Inhibit the growth of transplanted tumors	Validation was performed using only one ferroptosis inhibitor, without the use of an Nrf2 agonist	Liu et al. (2023)
			75,150 mg/kg	Naked mouse xenograft model				
Penexanthone A	*Candus ilicifolius L*.	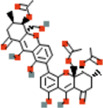	0.1.10 μg/mL	*In vitro* models of HCT116 and HT29 cells and *in vivo* xenograft models in zebrafish	GSH↓SOD↓HO-1↓SLC7A11↓GPX4↓	Significantly reduced tumor fluorescence intensity and proliferation area	Limited to cell and zebrafish models; lacks validation of efficacy in mammals	Zhao et al. (2024)
S-allylmercaptocysteine	*Allium sativum L.*	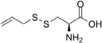	200,400 μM	HCT-116 cells	LC3-II↑Bax/Bcl-2↑p53↑p62↓	Significantly inhibits tumor growth	No pharmacokinetic or long-term safety evaluations have been conducted.	Li et al. (2017)
			300 mg/kg	Naked mouse xenograft model				
Cinnamyl aldehyde	*Cinnamomum verum J.Presl*	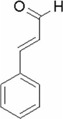	5–20 μmol/L	HCT116 cells	Ki-67↓COX-2↓ODC↓8-oxo-dG↓	Exerts an anti-colitis-associated colorectal cancer effect via Nrf2-dependent pathways	No human pharmacokinetic studies have been conducted	—
			0.1%,0.5% (w/w)	AOM/DSS-induced colitis-associated colorectal cancer model				
Carboxymethyl pachyman	*Poria cocos (Schw.) Wolf*	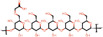	50,100 mg/kg	CT26 mice colon cancer xenograft model	CAT↑GSH-Px↑GSH↑ROS↓	Improves pathological damage to colonic tissue and reduces the mucosal injury score	The dose-response relationship has not been established, and there is a lack of *in vitro* cellular experiments to validate key pathways.	Wang K. et al. (2018)
Citraconate	*Morus alba L.*	------	12 mM	HCT116,MC38 cells	NQO1↓GCLC↓GCLM↓MDA↓	Inhibition of ferroptosis drives the progression of CRC	A lack of clinical cohort studies to validate its association with patient prognosis	Mai et al. (2024)
Brusatol	*Coptis chinensis Franch.*	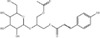	15–240 nmol/L	HCT116,HT29 cells	G6PD↓6GPD↓IDH1↓ME1↓	Significantly inhibits cell proliferation	Lack of *in vivo* animal models and clinical studies to validate the findings	Liu et al. (2018)

**TABLE 2:** Herbal compound regulate Nrf2 signaling pathway to treat CRC.

Extract	*Origination*	Dosage	Model	Biological effects	Results	Limitations	References
SYD	*Areca catechu L.* *Rheum palmatum L.* *Angelica sinensis (Oliv.) Diels* *Glycyrrhiza uralensis Fisch.* *Paeonia lactiflora Pall.* *Dolomiaea costus (Falc.) Kasana and A.K.Pandey* *Neolitsea cassia (L.) Kosterm.* *Coptis chinensis Franch.* *Scutellaria baicalensis Georgi*	0–8 mg/mL	HT-29 cells	NF-α↓IL-1β↓Ki-67↓HO-1↑NQO-1↑	Significantly improved phenotypic changes in the model mice, including weight loss, colon shortening, and an increase in tumor count	The core active ingredient of SYD has not been identified; no dose-response or clinical translation studies have been conducted.	Wang et al. (2020)
		18.5 g/kg	A mouse model of colorectal cancer associated with AOM/DSS-induced colitis				
QFG	*Scleromitrion diffusum (Willd.) R.J.Wang*, *Scutellaria barbata D. Don* *Astragalus mongholicus Bunge* *Hordeum vulgare L*.	0.5,1.2 mg/mL	HCT-116 cells	Nrf2↓SLC7A11↓GPX4↓ROS↑MDA↑	Inhibit cell proliferation	No *in vivo* animal studies or clinical sample validation have been conducted.	Wu et al. (2026)
Xihuang Capsules	*Calculus Bovis* *Moschus Artifactus* *Boswellia sacra Flück.*, and *Commiphora myrrha (T.Nees) Engl*.	5.28%	HCT-116 cells	BCL2↓MMP2↓MMP9↓ZNF320↓VEGFA↓GSK3β↓	Inhibits cell proliferation, promotes apoptosis, and reduces migration and invasion capabilities	These results are based solely on *in vitro* cell experiments and have not been validated in animal models or clinical trials.	Liang et al. (2024)
FSDQ	*Paeonia lactiflora Pall.* *Glycyrrhiza uralensis Fisch.* *Aconitum carmichaelii Debeaux* *Scutellaria baicalensis Georgi* *Sophora flavescens Aiton* *Rehmannia glutinosa (Gaertn.) Libosch. ex DC*.	0.25,0.5.1 mg/mL	CT-26 cells	Fe^2+^↑ROS↑MDA↑SOD↓Nrf2↓SLC7A11↓GPX4↓	Dependency inhibits cell proliferation and migration	The study relied solely on single-cell and subcutaneous tumor models and lacked *in situ* and clinical validation.	Zheng et al. (2024)
		4.49,8.97,17.94 g/kg	Mouse xenograft model				
ZJP	*Coptis chinensis Franch.* *Tetradium ruticarpum (A.Juss.) T.G.Hartley*	50 μg/mL	HCT116,Lovo,SW620 cells	ROS↑Fe^2+^↑GSH↓SLC7A11↓GPX4↓Nrf2↓MDM2↓	Inhibit cell proliferation	Only *in vitro* cell experiments have been conducted; validation using *in vivo* animal models is lacking.	Wei et al. (2023)

### 5.1 Flavonoids

Flavonoids are a class of polyphenolic secondary metabolites widely distributed in the plant kingdom. Their unique benzopyranone skeleton and diverse substituent groups confer a wide range of physiologically active functions. Studies have shown that flavonoids can inhibit CRC cell proliferation, promote apoptosis, arrest the cell cycle, and alleviate oxidative damage and inflammatory responses, while simultaneously inhibiting tumor invasion, metastasis, and angiogenesis, demonstrating significant research value and application potential in the prevention and intervention of CRC.

Ononin is a flavonoid compound isolated from the dried roots of *A. membranaceus* (Fisch.) Bge, possessing pharmacological activities such as antioxidant and anti-inflammatory effects (Sharma et al., 2024). Gui et al. systematically elucidated its anti-CRC effects and molecular mechanisms through network pharmacology and *in vitro* and *in vivo* experiments. *In vitro*, treatment of human CRC HCT116 and mouse CRC MC38 cells with Ononin (2–16 μM) for 24–48 h significantly inhibited cell proliferation, migration and invasion, and arrested the cell cycle in the G1 phase. Furthermore, Ononin inhibited PI3K phosphorylation, AKT phosphorylation and Nrf2 nuclear translocation, thereby suppressing cellular antioxidant capacity and Nrf2 nuclear translocation, promoting the massive generation of total intracellular ROS and lipid ROS, and exacerbating OS. Simultaneously, it reduced GSH levels and mitochondrial membrane potential, downregulated GPX4 expression, and induced ferroptosis via the PI3K/AKT/Nrf2 pathway. *In vivo*, the combination of oral administration of Ononin (60 mg/kg) and intraperitoneal injection of 3.5 mg/kg anti-PD-L1 demonstrated synergistic inhibition of tumour growth, increased the proportion of tumour-infiltrating IFN-γ^+^CD8^+^ effector T cells, and enhanced anti-tumour immunity without significant organ toxicity (Gui et al., 2025). However, Ononin has limitations including poor oral bioavailability and the lack of clearly defined biomarkers for clinical translation.

Tectorigenin (TEC), primarily derived from iridaceae plants such as *Iris domestica* (L.) Goldblatt and Mabb., possesses various pharmacological properties, including anti-inflammatory, antioxidant, antitumor, and cardioprotective effects (Rong et al., 2023). Using Caco-2 CRC cells and CT26 tumor-bearing mice as models, Ma et al. investigated the therapeutic effects of TEC combined with irinotecan (CPT-11) on CRC and the protective mechanisms against chemotherapy-induced intestinal injury. *In vitro* results showed that TEC (50, 100 μmol/L) significantly improved CPT-11- and LPS-induced barrier damage in Caco-2 cells by upregulating the expression of tight junction proteins such as ZO-1, occludin, and E-cadherin, reduced levels of IL-1β, IL-6, and TNF-α, and increased IL-10 levels, while simultaneously activating the Nrf2/Keap1 and promoting Nrf2 nuclear translocation. Nrf2 silencing reversed these effects, confirming that this pathway is a key mediating mechanism. *In vivo* experiments demonstrated that the combination of TEC (40, 80 mg/kg) and CPT-11 (50 mg/kg) alleviated weight loss, diarrhea, and colonic shortening in mice, improved intestinal pathological damage, suppressed colonic inflammation, and enhanced intestinal barrier function; simultaneously, it synergistically inhibited the growth of CT26 xenografts, promoted tumor cell apoptosis, and suppressed proliferation, without increasing the expression of drug-resistant efflux pumps (Ma et al., 2025). However, this study has some limitations, such as the lack of a systematic evaluation of the long-term safety of TEC on normal intestinal cells and tissues, and the failure to clarify the differential regulatory effects of Nrf2 in tumor and normal tissues.

Quercetin, a flavonoid compound widely found in *Styphnolobium japonicum* (L.) Schott and *Hypericum perforatum* L., possesses a broad range of pharmacological effects, including free radical scavenging, anti-inflammatory, and antitumor activities (Deepika and Maurya, 2022). Tang et al. demonstrated through *in vitro* cell experiments that the combination of quercetin and 5-FU could significantly reverse chemotherapy resistance in HCT116 CRC cells and their 5-FU-resistant cell lines. *In vitro* studies indicated that treating cells with 5-FU (10 μM) and quercetin (10, 40 μM) individually or in combination for 72 h significantly inhibited cell proliferation, promoted apoptosis, and reversed chemotherapy resistance. Quercetin significantly inhibited ROS production and downregulated the activity of antioxidant enzymes SOD, CAT, GPx and GR. Simultaneously, it inhibited the expression of Nrf2, HO-1, SOD-1 and CAT proteins, thereby blocking the Nrf2/HO-1 antioxidant pathway. Furthermore, it inhibited NF-κB activation, regulated p-IKBα expression, and lifted tumour anti-apoptotic protection (Tang et al., 2023). This study has only been validated at the cellular level and lacks support from *in vivo* animal models, clinical samples, and multiple cell lines. No pharmacokinetic, toxicological, or optimal dosage studies have been conducted, and the evidence for clinical translation remains insufficient.

Taxifolin (TAX) is a beneficial natural compound found in *Pseudotsuga menziesii* (Mirb.) Franco that possesses both antitumour and cytoprotective properties which plays a significant role in reducing chemotherapy-induced damage and inhibiting the progression of cancer (Li C. X. et al., 2022). In DMH-induced mouse CRC models, oral administration of TAX (30 mg/kg) markedly improved histopathological damage to the colon tissue, reduced serum CEA and LDH levels, and decreased mast cell infiltration, with a protective effect superior to that of 5-FU. TAX promoted Nrf2 nuclear translocation and the expression of antioxidant genes, thereby enhancing antioxidant capacity. It also inhibited NF-κB activation and the expression of TNF-α and COX-2, thereby improving the tumour inflammatory microenvironment. Simultaneously, it inhibited β-catenin nuclear translocation, downregulated Cyclin D1 and c-Myc, and prevented abnormal cell proliferation. TAX also activated the endogenous mitochondrial apoptosis pathway, promoting the expression of cytochrome C, caspase-9, caspase-3 and cleaved PARP, thereby inducing apoptosis in CRC cells (Manigandan et al., 2015). However, current research remains predominantly based on animal models, and further investigation is required regarding pharmacokinetics, dose-response relationships and clinical translation which could provide key evidence for the development of TAX for the chemoprevention of CRC.

Cyanidin chloride (CyCl) is a typical anthocyanin widely found in purple-red fruits and vegetables such as *Vaccinium corymbosum* L. and *Vitis vinifera* L., and possesses potent antiproliferative, anti-inflammatory and antioxidant activities (Safdar et al., 2023). Research by Lee et al. has confirmed that CyCl exerts significant antitumour effects on CRC cells, with its molecular mechanism closely linked to the cross-regulation of Nrf2 and NF-κB. *In vitro* experiments have confirmed that CyCl (10–100 μM) could induce apoptosis in CRC cells via the intrinsic mitochondrial apoptosis pathway, significantly upregulating Bax, cleaved caspase-3 and cleaved PARP, whilst downregulating anti-apoptotic proteins such as Bcl-2 and XIAP, and simultaneously inhibiting cell colony formation and malignant proliferation. In terms of signal regulation, CyCl blocked the phosphorylation of IκBα and IKKα/β, inhibited the nuclear translocation of p65/p50, and downregulated inflammatory factors such as TNF-α and IL-6, thereby inhibiting the NF-κB pathway. Simultaneously, it activated the Nrf2 pathway, promoted the nuclear translocation of Nrf2, and upregulated antioxidant proteins such as HO-1 and NQO1. Silencing Nrf2 reversed CyCl’s inhibitory effect on NF-κB and its pro-apoptotic effects, confirming that Nrf2 is a key upstream node in its anticancer action (Lee et al., 2020). In summary, CyCl exerts its anti-CRC effects by activating Nrf2 to inhibit NF-κB, demonstrating potential translational value. However, evidence regarding direct upstream targets, *in vivo* animal validation and the intestinal microenvironment is currently lacking, and further mechanistic and clinical studies are required.

Formononetin (FN) is an active compound extracted from *Astragalus mongholicus* Bunge that has garnered widespread attention for its anti-inflammatory, antioxidant, and antitumor activities (Aliya et al., 2023). Tang et al. established a CRC model in BALB/c mice using CT26 cells and combined it with 10 Gy abdominal irradiation to create a radiation-induced intestinal injury (RIII) model, thereby confirming the protective effects of FN. *In vivo* experiments demonstrated that FN (20 mg/kg) significantly enhanced small intestinal absorption function in mice, improved histopathological damage to intestinal tissue, and upregulated the expression of tight junction proteins Claudin-1, Occludin, and ZO-1 to repair the intestinal mechanical barrier; it also reduced intestinal tissue apoptosis rates and Cleaved-caspase 3 levels, promoted CD31-mediated angiogenesis, and simultaneously reduced ROS and NO production, thereby alleviating OS. Mechanistically, FN exerted its protective effects by downregulating cytoplasmic Keap1 and Nrf2 expression, promoting Nrf2 nuclear translocation and activation, and activating Keap1-Nrf2 (Tang et al., 2025). However, this study did not investigate the direct binding of FN to Keap1, nor did it include a normal mouse radiotherapy control group; therefore, the multi-target synergistic mechanism and clinical translation potential require further validation.

Dihydromyricetin (DMY) is a natural flavonoid compound extracted from plants of the *V. vinifera* L., possessing anti-inflammatory, antioxidant and broad-spectrum antitumour activity (Wu et al., 2022). Wang et al. conducted *in vitro* experiments using DMY in oxaliplatin (OXA)-resistant CRC cells (HCT116/L-OHP) and their parental HCT116 cells, confirming its ability to reverse multidrug resistance and enhance the antitumour activity of oxaliplatin. *In vitro* experiments demonstrated that DMY (25, 50, 100 μmol/L) synergised with oxaliplatin to inhibit the proliferation of resistant cells and significantly increased apoptosis levels via the intrinsic mitochondrial apoptosis pathway, whilst not affecting the drug sensitivity of the parental cells (Wang et al., 2017). DMY specifically inhibited the expression and efflux function of MRP2, a key protein in drug resistance, thereby increasing intracellular drug accumulation. Its core regulatory pathway involves blocking Nrf2 nuclear translocation, which in turn inhibits the transcriptional activation of MRP2 by Nrf2/ARE, ultimately reversing MRP2-mediated multidrug resistance. Current research is limited to the cellular level. *In vivo* efficacy, safety and other potential targets remain to be elucidated, and further validation through animal studies and systematic preclinical research is required.

### 5.2 Terpenoids

Terpenoids are a class of natural secondary metabolites widely found in nature, composed of isoprenyl units. They exhibit a rich variety of structural types and highly variable skeletal frameworks, thereby possessing diverse and prominent biological activities. Studies have shown that terpenoids can effectively inhibit CRC cell proliferation, induce tumor cell apoptosis, and arrest the cell cycle; simultaneously, they can regulate inflammation-related pathways, inhibit invasion, metastasis, and angiogenesis, demonstrating significant theoretical significance and application potential in CRC prevention and intervention research.

Tagitinin C (TC), a bioactive metabolite derived from the subtropical medicinal plant *Tithonia diversifolia* (Hemsl.) A. Gray, exhibits clear antitumour activity in CRC. It significantly inhibits the proliferation and migration of CRC cells and induces cell cycle arrest, demonstrating potential for development as a candidate drug against CRC (Andrade et al., 2026). Through *in vitro* experiments, Wei et al. found that TC (5, 10, 20 μM) inhibited the proliferation and migration of SW480, DLD1, and HCT116 cells in a concentration- and time-dependent manner, whilst inducing cell cycle arrest. TC (20 μM) triggered early ferroptosis in HCT116 cells. Mechanistically, TC promoted PERK phosphorylation and eIF2α activation, accompanied by Nrf2 nuclear translocation and HO-1 upregulation. Meanwhile, it triggered robust ROS generation and inhibited the NF-κB, TNF-α and COX-2 pathways. The above changes increased LPO, MDA content and labile iron pool, depleted intracellular GSH, and reduced mitochondrial membrane potential. All these effects ultimately induced ferroptosis (Wei et al., 2021). The combination of TC (10 μM) and Erastin (20 μM) exhibited a synergistic anti-tumour effect, significantly enhancing ferroptosis. Current research is limited to *in vitro* studies and further investigation is required regarding *in vivo* efficacy, safety, pharmacokinetics, and the underlying mechanisms in drug-resistant tumours.

Triptolide (TPL), the principal active component extracted from *Tripterygium wilfordii* Hook. f., has attracted considerable attention due to its anti-inflammatory and antitumour pharmacological properties (Song et al., 2019). Wang et al. used TPL as the test compound to investigate its effects on inhibiting the proliferation and invasion of CRC cells, as well as the underlying molecular mechanisms, in human CRC HT29 cell models. *In vitro* experiments involved treating HT29 cells with TPL at concentrations of 0, 25, 50 and 100 nmol/L for 24 h. The results showed that TPL inhibited cell proliferation, promoted apoptosis and reduced invasive capacity, with the most significant effect observed at a concentration of 100 nmol/L. TPL exerted its effects primarily via the intrinsic mitochondrial apoptosis pathway, promoting the release of Cytochrome C, the activation of caspase-9 and caspase-3, the generation of cleaved PARP, and DNA fragmentation. It also inhibited Nrf2 protein expression and pathway activation, downregulated Nrf2-downstream pro-proliferative signals, and further reduced MMP-2 and MMP-9 levels, thereby significantly impairing cell invasion and metastasis capacity. Following Nrf2 silencing, the antiproliferative, pro-apoptotic and anti-invasive effects of TPL were further enhanced, confirming that TPL exerts its anti-CRC effects by blocking Nrf2 and regulating MMP-2/9 (Wang Y. et al., 2024). This study was conducted using *in vitro* cell experiments; *in vivo* validation and validation of upstream and downstream molecules in the pathway have not yet been carried out.

Research by Liang et al. confirmed that Astragaloside IV (AS-IV) could significantly block the development of colitis-associated CRC induced by AOM/DSS in mice. AS-IV (20, 40, 80 mg/kg) exerted protective effects on intestinal inflammation. It improved intestinal microenvironment, attenuated colonic mucosal lesions and tissue structural disorder, and blocked tumor formation and abnormal cell growth. In addition, AS-IV downregulated pro-inflammatory factors IL-1β, IL-6 and TNF-α. The PPARγ-Nrf2/HO-1 antioxidant axis was activated, accompanied by the restoration of antioxidant function mediated by SOD, CAT and GSH. Decreased ROS and NO accumulation alleviated OS and subsequent DNA damage. *In vitro* experiments further confirmed that AS-IV (5, 20, 50 μM) could directly bind to and activate PPARγ, reverse LPS-induced intestinal epithelial inflammation and DNA breaks which could be blocked by the PPARγ inhibitor GW9662 (Liang et al., 2023). This study clearly demonstrates that the anti-CAC effect of AS-IV relies on PPARγ-mediated antioxidant, anti-inflammatory and DNA-protective mechanisms. However, its impact on the polarisation of intestinal immune cells remains to be elucidated, and the positive feedback regulation between PPARγ and Nrf2 requires further validation.

Limonin is a class of naturally occurring tetracyclic triterpenoid secondary metabolites found in plants of the Rutaceae and Meliaceae families like *Citrus aurantium* f. Aurantium, and is one of the primary components responsible for the bitter taste in citrus fruits. In mouse models of colorectal adenocarcinoma induced by combination of AOM and DSS, intervention with Limonin (50 mg/kg) significantly improved weight loss, shortened colon length and abnormal spleen weight. It effectively reduced tumour incidence and disease activity index, and alleviated the degree of colonic mucosal inflammatory infiltration, glandular structural disruption, dysplasia and adenocarcinoma lesions. It also brought the overall phenotype closer to that of normal tissue, suggesting that limonin exerts a clear inhibitory effect on inflammation-associated colorectal adenocarcinoma. Mechanistic studies revealed that Limonin promoted PPARγ protein binding, nuclear translocation of PPARγ, and PPARγ-Nrf2 positive feedback activation. It significantly reduced MDA and PGE2 levels, downregulated TNF-α gene expression in colonic tissue, whilst simultaneously upregulating Nrf2 and SOD2 transcription levels and increasing GSH content. By enhancing the body’s antioxidant defence system and mitigating OS damage and chronic inflammatory responses, it exerted a protective effect on the gut (Ishak et al., 2021). This study has certain limitations, such as the use of a single dose, a small sample size, and short-term animal experiments. No long-term toxicity evaluation, metabolic stability analysis, or human clinical trials have been conducted. Consequently, the safety and clinical translational feasibility of limonin for long-term intervention in colorectal adenocarcinoma cannot yet be fully substantiated.

### 5.3 Glycosides

Glycosides are a class of natural secondary metabolites widely found in plants, formed by the condensation of a sugar moiety and a non-sugar component. They exhibit diverse structural types and a wide variety of glycosidic bond configurations, demonstrating broad and significant biological activity. Studies have shown that glycosides can inhibit the proliferation of CRC cells, induce apoptosis, arrest the cell cycle, and regulate inflammatory and OS pathways, while simultaneously inhibiting tumor invasion and metastasis, demonstrating significant potential for application in the prevention and intervention of CRC.

Ginsenoside Rh3 is an active saponin extracted from the roots, stems and leaves of *Panax ginseng* C.A.Mey (family Araliaceae), possessing potent anti-inflammatory and anti-tumour effects (Cong et al., 2020). Research by Wu et al. confirmed its significant anti-cancer effects on CRC: *in vitro*, HT29 and HCT116 CRC cells were treated with Ginsenoside Rh3 (10–160 μM); *in vivo*, nude mice with transplanted tumours were administered Ginsenoside Rh3 (20 mg/kg/day) via oral gavage. The results showed that Ginsenoside Rh3 inhibited tumour cell proliferation, reduced clonogenicity, and significantly decreased the volume and weight of transplanted tumours, whilst exhibiting no significant toxicity to normal colon cells or the liver and kidneys. Ginsenoside Rh3 acted by regulating the Stat3/p53/NRF2 axis, inhibiting Stat3 phosphorylation and suppressing Nrf2 nuclear translocation. On the one hand, it inhibited Nrf2 nuclear translocation, downregulated HO-1, suppressed GSH and the antioxidant defence system, and activated the NLRP3/caspase-1/GSDMD pathway to induce pyroptosis. On the other hand, it inhibited SLC7A11 (xCT), GPX4, GSH, thereby promoting iron accumulation, the generation of lipid ROS, elevated MDA levels and LPO to trigger ferroptosis, whilst also promoting the release of IL-1β and IL-18 to modulate the tumour immune microenvironment (Wu Y. et al., 2023). This study confirms that it exerts anti-CRC effects through the synergistic action of pyroptosis and ferroptosis. However, validation has been limited to cellular and nude mouse models, with a lack of evidence from large animal models and clinical translation. *In vivo* pharmacokinetics and clinical safety require further investigation.

Amarogentin is a furanocoumarin glycoside compound extracted from plants of the Gentianaceae family (such as *Centaurium erythraea* Rafn and *Dendrobium wentianum* J.J.Sm.), possessing various biological activities including anti-inflammatory, antioxidant and anticancer properties (Zhang et al., 2020). Anti-CRC effects and molecular mechanisms in in vitro CRC cells, patient-derived tumour-like cell clusters (PTCs) and *in vivo* nude mouse xenograft models. *In vitro*, using HCT116, HT-29 cells and PTCs as models, AG (20–160 μg/mL) was administered for 12–72 h to observe its effects on cell proliferation, ferroptosis and invasion and metastasis. *In vivo*, subcutaneous tumour xenograft models were established in nude mice. AG (0.2 μg/g/d) was administered via continuous oral gavage for 28 days, with 5-FU serving as a positive control. Results showed that AG inhibited cell proliferation, promoted the massive generation of ROS, elevated MDA levels, induced Fe^2+^ accumulation and LPO, and suppressed GPX4 activity and the cellular antioxidant defence system. By inducing ferroptosis, it significantly inhibited cell migration, invasion and the EMT process (Wang et al., 2025). Mechanistically, AG downregulated the mRNA and protein expression of Nrf2, HO-1 and GPX4, thereby blocking the activation of this pathway. *In vivo* experiments confirmed that AG significantly reduced tumour size and Ki-67 levels, demonstrating a marked antitumour effect. This study demonstrates that AG exerts its anti-CRC effects by inducing ferroptosis, inhibiting the Nrf2/HO-1/GPX4 pathway and EMT, providing experimental evidence for subsequent research and development. However, further clinical validation, combination therapy and safety studies are still required.

### 5.4 Alkaloid compounds

Alkaloids are a diverse class of nitrogen-containing bioactive compounds derived from plant secondary metabolites, whose pharmacological activity is primarily determined by the nitrogen-containing heterocyclic skeletons and functional group substitutions within their molecules. These compounds can inhibit tumor cell proliferation, induce apoptosis, arrest the cell cycle, modulate the tumor immune microenvironment, and interfere with inflammatory pathways, demonstrating promising applications and broad development potential in CRC prevention and treatment research.

*In vitro* CRC cell models and AOM/DSS-induced mouse models, Delavinone has demonstrated significant anti-CRC effects. *In vitro* experiments indicated that Delavinone (200 μM) effectively inhibited the proliferation of HT29 and HCT116 cells. By triggering OS, elevating levels of lipid ROS and MDA, and depleting GSH, it specifically induceed ferroptosis in tumour cells, accompanied by the activation of endogenous mitochondrial apoptosis, thereby further enhancing the cytotoxic effect. *In vivo* administration of Delavinone (2.5, 10 mg/kg) as a continuous intervention significantly improved pathological phenotypes in model mice, such as weight loss and colonic shortening, reduced tumour burden and Ki-67 levels, and alleviated intestinal tissue damage. Mechanistically, Delavinone directly targeted and inhibited PKCδ kinase activity, blocked Nrf2 phosphorylation and nuclear translocation, and downregulated antioxidant target genes such as GPX4 and SLC7A11, thereby driving ferroptosis via the PKCδ/Nrf2/GPX4 signalling axis (Zhang et al., 2025). This study confirms that Delavinone is a novel ferroptosis inducer, providing a new candidate for CRC drug development. However, the study has not yet clarified its long-term safety, pharmacokinetics, or potential for clinical translation. Normal tissue toxicity, *in vivo* metabolism, and clinical application value still require further systematic investigation.

Piperlongumine (PL) is a natural alkaloid extracted from *Piper longum* L., a plant of the Piperaceae family, and possesses significant antitumour and chemotherapeutic sensitising effects. A study elucidated the molecular mechanism by which PL reverses 5-FU resistance in human CRC HCT-8/5-FU cells and validated its action pathways and targets through *in vitro* and *in vivo* experiments. *In vitro*, PL (5 μM) induced massive ROS accumulation by specifically inhibiting thioredoxin reductase (TrxR), thereby inhibiting Akt phosphorylation and modulating the Akt/FoxO3/Nrf2 signalling axis. PL also promoted Bad dephosphorylation, cytochrome C release, activation of caspase-9 and caspase-3, generation of cleaved PARP, DNA fragmentation and p53 upregulation, thereby potently activating endogenous mitochondrial apoptosis, meanwhile inhibiting Nrf2 activation, P-gp expression and cyclin D1-related proliferation proteins, reducing drug efflux and arresting the cell cycle. *In vivo* tumour xenograft studies in nude mice demonstrated that PL (2.5 mg/kg) in combination with 5-FU (20 mg/kg) synergistically inhibited tumour growth (Zhou et al., 2025). This study remains limited: it utilised only a single strain of drug-resistant cells and lacked validation across multiple models. No long-term toxicity or pharmacokinetic studies of the combination were conducted, and further evidence is required to support clinical translation.

Cepharanthine (Cep) is a natural alkaloid extracted from the roots of the *Stephania japonica* (Thunb.) Miers, and possesses significant anticancer activity (Lu et al., 2024). Li et al. experimentally demonstrated that Cep (5, 10, 20 μM) could specifically inhibit TOM20 and TOM70, which are highly expressed in CRC cells, thereby disrupting mitochondrial structure and membrane potential. This subsequently inhibited the Nrf2 antioxidant pathway, downregulated antioxidant proteins such as GPX4, SOD and CAT, whilst simultaneously promoting the expression of Bax and Cleaved-caspase-3, thereby initiating apoptosis-related signalling. Cep also exacerbated the accumulation of ROS within cells, promoted iron accumulation and LPO, and upregulated ferroptosis markers such as TfR-1, ACSL4 and COX-2, ultimately driving ferroptosis. Replenishing TOM70 or using the ferroptosis inhibitor Fer-1 could significantly reverse its anticancer effects (Li et al., 2024). This study confirms that Cep exerts its anti-CRC effects via the TOM/NRF2/ferroptosis axis, providing a new direction for targeted therapy with natural products. However, *in vivo* experiments and precise validation of targets are still required to advance clinical translation. What’s more, the study was limited to *in vitro* cellular experiments and lacked validation in in vivo animal models. Furthermore, precise interventions such as TOM70 gene knockout or site-specific mutations were not performed. Consequently, the specificity of the target and the applicability for clinical translation require further refinement.

Aloperine (ALO) is a quinolizidine alkaloid extracted from the *Lablab purpureus* (L.) Sweet. This study confirmed that ALO (10–800 μM) inhibited the proliferation of HCT116 and SW480 CRC cells in a concentration-dependent manner. ALO (200, 300 μM) significantly activated the intrinsic mitochondrial apoptosis pathway, promoting the expression of Bax, Caspase-9 and Caspase-3, as well as the release of Cyt-C, and facilitating the assembly of the mitochondrial apoptosis complex. Concurrently, it inhibited Bcl-2 and reduced the Bcl-2/Bax ratio, thereby efficiently inducing apoptosis. Furthermore, ALO promoted LPO, elevated MDA levels, intracellular Fe^2+^ accumulation and massive ROS production, reduced GSH levels, and induced ferroptosis. DFO (20 μM) and Fer-1 (10 μM) could reverse these effects. ALO directly bound to the Nrf2 protein, inhibited Nrf2 expression, nuclear translocation, and the downstream antioxidant/anti-ferroptosis axis, thereby downregulating key proteins such as GPX4, xCT, and DMT1. Overexpression of Nrf2 significantly attenuated the anticancer effects of ALO (An et al., 2026). This study has only completed *in vitro* validation and lacks *in vivo* animal experiments, pharmacokinetic and toxicological data. In summary, ALO simultaneously drives apoptosis and ferroptosis by targeting Nrf2, demonstrating good potential for combating CRC and warranting further translational research.

In 5-FU resistance CRC models induced by a tumour hypoxic microenvironment, Kim et al. treated various CRC cell lines including HCT116 and HT29, with Quinacrine (QC, 0–5 μM) alone or in combination with 5-FU (0–5 μM). *In vivo* experiments involved oral administration of QC (100 mg/kg) combined with intraperitoneal injection of 5-FU (5 mg/kg) to treat HCT116 cell xenograft models in nude mice. The results indicated that the combination of QC and 5-FU significantly reversed hypoxia-mediated 5-FU resistance, enhanced the cytotoxic effects of chemotherapy, and effectively inhibited tumour growth *in vivo* (Kim et al., 2019). The mechanism suggests that QC activates JNK1, inhibits the stability of the downstream Nrf2 protein, promotes Nrf2-Keap1 binding, Cul3-dependent ubiquitination and proteasomal degradation, whilst not affecting Nrf2 transcription levels. This promotes DNA double-strand breaks (DSBs), γ-H2AX, TUNEL-positive apoptotic bodies and caspase cascade activation, significantly promoting apoptosis in tumour tissue, Nrf2 degradation and enhancing the antitumour effects of 5-FU. This combination regimen offers a new strategy for sensitising CRC to chemotherapy. However, validation has been limited to preclinical cell and animal studies, with a lack of human pharmacokinetic, safety and resistance mutation data. Clinical translation requires further support from clinical trials.

### 5.5 Phenolic compounds

Phenolic compounds, as a class of important secondary metabolites widely found in plants, exhibit biological activities that are closely related to their aromatic ring structures and the substitution patterns of their phenolic hydroxyl groups. These compounds exert anticancer effects through multiple mechanisms, including inhibiting CRC cell proliferation, promoting tumor cell apoptosis, blocking abnormal cell cycle progression, modulating the tumor immune microenvironment, and alleviating chronic inflammation and OS damage. They have demonstrated significant theoretical value and potential for clinical translation in CRC prevention and treatment research.

Curcumin is a phenolic compound extracted from the roots and rhizomes of turmeric (*Curcuma longa* L.), possessing pharmacological properties such as anti-inflammatory and antioxidant effects. Liu et al. found through experiments that *in vitro*, curcumin (15 μmol/L) could activate the endogenous mitochondrial apoptosis pathway, promote the expression of Bax, cleaved caspase-3,cleaved PARP, inhibit Bcl-2 and reduce the Bcl-2/Bax ratio, whilst simultaneously inducing senescence, causing G0/G1 phase arrest in p53 wild-type cells and G2/M phase arrest in p53-deficient cells, thereby significantly inhibiting the proliferation of CRC cells (Liu et al., 2023). Furthermore, curcumin increased intracellular ROS levels, inhibited Keap1-mediated cytoplasmic anchoring and degradation of Nrf2, promoted Nrf2 nuclear translocation and NQO1 expression, enabling Nrf2 to directly bind to the miR-34a/b/c promoters and upregulate their transcription. This reversed the suppression of the miR-34 family by hypoxia and IL-6, thereby downregulating vimentin, SNAIL, SLUG and ZEB1 to block EMT, thereby inhibiting cell migration and invasion. Although curcumin exerted its anticancer effects via the ROS/KEAP1/Nrf2/miR-34a/b/c cascade, its clinical translation remains limited by issues such as low *in vivo* bioavailability and insufficient targeting and accumulation efficiency. And there is a lack of validation from large-scale clinical studies. Nanodelivery and formulation optimisation strategies may provide key support for its clinical application.

Epigallocatechin gallate (EGCG) is the most abundant natural polyphenolic active component in green tea, possessing significant pharmacological effects including antioxidant, anti-inflammatory and antitumour properties (Capasso et al., 2025). Enkhbat et al. treated human CRC HCT116 cells with a combination of EGCG (12.5 μmol/L) and 2 Gy X-rays, confirming that this significantly enhanced the radiosensitivity of tumour cells. This combined treatment significantly promoted Nrf2 nuclear translocation, whilst upregulating LC3 mRNA expression and caspase-9 mRNA expression, and activating the caspase-9 and LC3-mediated autophagy-apoptosis linkage pathway, synergistically inhibiting cell proliferation and colony formation, with a significantly superior anti-tumour effect compared to monotherapy. The mechanism suggested that EGCG enhanced the radiotoxic effect by promoting Nrf2 nuclear translocation, upregulating LC3 and caspase-9 expression, and activating the Nrf2/autophagy signalling axis to initiate endogenous apoptosis (Enkhbat et al., 2018). Currently, this has only been validated at the *in vitro* cellular level. Further research is required to investigate *in vivo* efficacy, safety, and the precise regulatory mechanisms of these pathways.

In a recent study, Li et al. utilised HCT116 and SW480 CRC cell lines to investigate the anti-CRC mechanism of hydroxytyrosol (HT). *In vitro* experiments demonstrated that, within the concentration range of 50–200 µM in HCT116 cells and 40–320 µM in SW480 cells, HT exhibited dose- and time-dependent inhibition of tumour cell proliferation, colony formation and migration capacity, whilst also blocking the cyclin D1-related pathway. HT promoted iron accumulation, LPO, massive ROS production and Tfr1 upregulation, leading to mitochondrial ruffling and rupture. Simultaneously, it inhibited Nrf2 nuclear translocation, Nrf2 and NQO1 expression, and downstream antioxidant proteins, reduced GSH levels and mitochondrial membrane potential, ultimately inducing ferroptosis (Li et al., 2025). This study confirms that HT exerts its antitumour effects by inhibiting Nrf2 and driving ferroptosis, thereby providing a new candidate for drug-resistant CRC. However, the research is currently limited to *in vitro* studies and lacks data on *in vivo* efficacy, safety, and chronic toxicity. Further *in vivo* validation is required prior to clinical translation.

Ginnalin A is a natural polyphenolic compound isolated from *Acer tataricum subsp. Ginnala* (Maxim.) Wesm., exhibiting remarkable antioxidant, anti-inflammatory, and antitumor activities. Bi et al. utilized *in vitro* cellular models to elucidate the role and molecular mechanisms of Ginnalin A in CRC chemoprevention. The results indicated that Ginnalin A (20, 40, 80 μM) significantly inhibited the proliferation and colony formation of three CRC cell lines-HCT116, SW480, and SW620, with the inhibitory effect increasing with higher concentrations. At the cellular level, Ginnalin A (40, 80 μM) inhibited tumor cell proliferation by interfering with the cell cycle process, without inducing significant apoptosis within the aforementioned concentration range. Regarding the mechanism of action, Ginnalin A upregulated the expression of Nrf2, HO-1, and NQO1 in a concentration-dependent manner and promoted Nrf2 nuclear translocation. Concurrently, it downregulated Keap1 and upregulated p62, thereby activating Nrf2/HO-1 to exert chemopreventive effects (Bi et al., 2018). This study confirms that Ginnalin A inhibits CRC cells by targeting Nrf2, providing experimental evidence for its development as a candidate natural active compound for the prevention and treatment of CRC. However, this study is limited to *in vitro* validation; at a concentration of 80 μM, Ginnalin A exhibited abnormal trends in the expression of some proteins, and the optimal concentration still requires optimization. Furthermore, validation in animal models is lacking, and the *in vivo* efficacy and underlying mechanisms require further investigation.

Zhu et al. systematically elucidated the regulatory roles and molecular mechanisms of procyanidin B2 (PB2) in intestinal injury repair and tumourigenesis using mouse models of abdominal radiation-induced intestinal injury, DSS colitis, and colitis-associated tumours. *In vivo* studies showed that PB2 (30, 50, 100 mg/kg) significantly improved survival rates in irradiated mice, alleviated intestinal OS, and inhibited Bax- and cleaved caspase-3-mediated endogenous mitochondrial apoptosis, whilst simultaneously activating Nrf2/ARE to enhance antioxidant capacity (e.g., SOD) and maintain mitochondrial homeostasis. PB2 also activated Wnt/β-catenin in an Nrf2-dependent manner, upregulated the activity of Lgr5+ intestinal stem cells, promoted intestinal epithelial proliferation and crypt regeneration, and inhibited NF-κB p65 activation, thereby alleviating colonic inflammation and reducing tumour incidence and malignancy. *In vitro* experiments have confirmed that PB2 (20 μM) reduced ROS accumulation in normal colonic epithelial cells and promoted proliferation and budding in intestinal organoids. What’s more, it did not confer radiation protection to CRC cells, a phenomenon associated with its inability to stabilise the Nrf2 protein in cancer cells (Zhu et al., 2021). The above studies indicate that PB2 exerts its intestinal protective effects by regulating OS, stem cell regeneration and inflammatory pathways, making it a potential natural compound for the prevention and treatment of radiation-induced intestinal injury and inflammatory bowel disease. However, its precise targets, pharmacokinetics and formulation optimisation require further investigation.

### 5.6 Coumarin compounds

Coumarin compounds are widely distributed bioactive substances with a benzopyranone skeleton among plant-derived secondary metabolites. Their anti-CRC pharmacological activity is primarily determined by the benzopyranone core and the modifications of substituent groups within the molecule. Studies have shown that coumarin compounds can inhibit CRC cell proliferation, induce tumor cell apoptosis, arrest the cell cycle, modulate the tumor immune microenvironment, and interfere with tumor-associated inflammatory pathways, demonstrating promising applications and broad development potential in CRC prevention and treatment research.

Esculin is a hydroxycoumarin compound extracted from the stem bark of *Fraxinus chinensis* subsp. *Rhynchophylla* (Hance) A.E.Murray and possesses anti-inflammatory and antitumour effects (Li W. et al., 2022). Ji et al. utilised *in vitro* cell cultures and AOM/DSS mouse models to elucidate its anti-CRC mechanism. *In vitro* studies using CRC cell lines such as HCT116 demonstrated that esculin (20, 40, 80 μM) significantly inhibited the viability of these cells, promoted the expression of Bax, p53,CHOP, cleaved-caspase-3 and DNA fragmentation, thereby activating the intrinsic mitochondrial apoptosis pathway. Simultaneously, it promoted the massive production of intracellular ROS, LPO and elevated MDA levels, disrupting the antioxidant system and inducing OS. Esculin also promoted the activation of p-Nrf2 and HO-1, as well as Fe^2+^ overload, and induced high expression of COX2 and ACSL4, Fe^2+^ accumulation, and elevated MDA levels, thereby triggering ferroptosis. Its core mechanism involved the activation of the endoplasmic reticulum stress PERK/Nrf2/HO-1 axis, achieving synergistic cell death through apoptosis and ferroptosis (Ji et al., 2024). A 12-week *in vivo* intervention with esculin (20–40 mg/kg) improved weight loss in mice, reduced the number of colon tumours and alleviated pathological damage. Although this study elucidated a new mechanism and the application potential of esculin, it has limitations: the safety profile for normal intestinal cells has not been systematically evaluated, and the *in vivo* pharmacokinetics, bioavailability and metabolic pathways remain unclear.

Auraptene is a natural coumarin abundant in citrus plants (such as *Citrus aurantium f.* aurantium), possessing anti-inflammatory, antioxidant and broad-spectrum antitumour activity. Ebrahimi et al. demonstrated through *in vitro* and *in vivo* models of CRC that this compound exerted anticancer effects via OS imbalance and mitochondrial apoptosis pathways. *In vitro* experiments showed that auraptene (50, 100, 200 μM) significantly inhibited the proliferation of CT26 spheroids and rapidly elevated intracellular ROS levels. In mouse subcutaneous tumour xenograft models, following 14 days of intratumoural administration, auraptene (50, 100, 200 μM) reduced antioxidant capacity as measured by CAT, GSH and FRAP, increased MDA levels, and exacerbated oxidative damage in tumour tissue, simultaneously upregulating Bax and downregulating Bcl-2, the anti-apoptotic factor Survivin, Cyclin D1, and the antioxidant transcription factor Nrf2, ultimately inducing tumour cell apoptosis and triggering cell cycle arrest (Ebrahimi et al., 2023). Mechanistically, auraptene primarily exerts its antitumour effects by inhibiting Nrf2 and disrupting redox homeostasis, providing a natural candidate for adjuvant therapy in CRC. However, this study utilised only a single xenograft model and did not address combination therapy, pharmacokinetics or long-term safety and the correlation between *in vitro* and *in vivo* targets still requires further validation.

### 5.7 Polysaccharide compounds

Carboxymethyl Pachyman (CMP) is a water-soluble derivative of Poria cocos polysaccharides obtained through carboxymethylation, exhibiting anti-inflammatory, antioxidant and immunomodulatory effects. Wang et al. demonstrated in CT26 CRC-bearing mouse models that daily oral administration of CMP (50, 100 mg/kg) combined with intraperitoneal injection of 5-FU (25 mg/kg) for 14 consecutive days significantly improved 5-FU-induced colonic shortening and histopathological damage, and alleviated intestinal mucositis. The mechanism involved excessive ROS production, upregulation of CAT and GSH-Px activity and GSH levels, and activation of the Nrf2-ARE pathway to enhance antioxidant capacity. It downregulated NF-κB and p-p38 MAPK expression to alleviate intestinal inflammation. It also inhibited Bax and the Bax/Bcl-2 ratio, and upregulated Bcl-2 to exert an anti-apoptotic effect. Concurrently, CMP restored gut microbiota diversity, increased the abundance of beneficial bacteria such as *Bacteroides* and *Lactobacillus*, and elevated the levels of short-chain fatty acids (SCFAs) such as acetic acid and butyric acid. The above studies indicated that CMP exerts antioxidant, anti-inflammatory, anti-apoptotic and gut microbiota-regulating effects through the synergistic regulation of the NF-κB, Nrf2-ARE and MAPK/p38 (Wang K. et al., 2018). This study has certain limitations: only two CMP doses were tested, the dose-response relationship was not clearly established, there was a lack of *in vitro* cellular experiments to validate key pathways, long-term safety and toxicological evaluations were not conducted, and the mechanisms of mutual regulation between the gut microbiota and signalling pathways were not explored in depth.

### 5.8 Other classes of compounds

Angelic acid is a naturally occurring unsaturated carboxylic acid commonly found in plants such as *Angelica sinensis* (Oliv.) Diels, and possesses pharmacological properties including anti-inflammatory, antioxidant and anticancer effects. Cao et al. utilised *in vitro* cellular and *in vivo* animal models to investigate the anti-CRC effects and molecular mechanisms of angelic acid, establishing that it exerts anticancer effects by regulating ferroptosis. *In vitro* experiments demonstrated that angelic acid significantly induced ferroptosis in CRC cells, accompanied by enhanced LPO, malondialdehyde (MDA) accumulation, and upregulation of ferroptosis-associated molecules CHAC1 and PTGS2. Mechanistically, angelic acid directly bound to the Nrf2 protein, promoted its K48-ubiquitination and degradation, thereby reducing protein stability and lifting Nrf2 inhibitory effect on ferroptosis. In CT26 cell orthotopic tumour models, the combination of angelic acid and the ferroptosis inducer sulfasalazine synergistically inhibited tumour growth without causing significant organ toxicity in mice (Cao Y. et al., 2025). This study provides a new strategy for the antitumour activity of natural products, but limitations remain: the upstream regulatory factors of Nrf2 ubiquitination have not been identified, the methods for target validation are limited, and no comparison has been made with clinical chemotherapy drugs. Therefore, its potential for clinical translation requires further exploration.

Arenobufagin (Are) is a compound extracted and isolated from the secretions of *Bufo gargarizans*, which has been found to possess antitumour pharmacological effects (Long et al., 2024). Wang et al. utilised CRC cell lines and NOD/SCID mouse lung metastasis models to investigate the effects and molecular mechanisms by which Are inhibits lung metastasis in CRC. *In vitro*, Are (20–80 nM) inhibited CT26 cell proliferation and increased intracellular ROS levels. *In vivo*, treatment with Are (0.5, 1, 2 mg/kg/day) reduced antioxidant markers such as CAT and GSH in tumour tissue, elevated MDA levels, and disrupted redox homeostasis. Concurrently, Are inhibited key molecules involved in EMT and matrix degradation, such as β-catenin, MMP3 and MMP9. It also downregulated c-MYC, Nrf2, HO-1 and downstream metastasis-related proteins and upregulated Bax whilst downregulating Bcl-2, Survivin and Cyclin D1, thereby inducing apoptosis, triggering cell cycle arrest and inhibiting metastasis (Wang Y. et al., 2024). Its core mechanism involves inhibiting Nrf2 and disrupting redox balance, providing experimental evidence for adjuvant therapy in CRC. However, this study utilised only a single subcutaneous tumour xenograft model and did not conduct evaluations of combination therapy, pharmacokinetics, or long-term safety. The correlation between *in vivo* and *in vitro* targets still requires further validation.

To elucidate the anti-CRC effects and molecular mechanisms of Brassinin (BSN), Wen et al. conducted a systematic study utilising network pharmacology, cellular experiments and nude mouse xenograft models. *In vitro* experiments demonstrated that BSN (1–400 μM) inhibited the viability of RKO and HCT116 CRC cells, whilst exhibiting no significant toxicity to normal colonic epithelial NCM460 cells. BSN (200 μM) directly bound to Nrf2 and GPX4 proteins, inhibited GPX4 expression and the activation of p62/NRF2/HO-1, whilst simultaneously inhibiting Nrf2 activation, p62 expression, and HO-1 transcription and expression, thereby inducing LPO. The ferroptosis inhibitor DFO reversed this effect, confirming that BSN exerts its anticancer effects by inducing ferroptosis. *In vivo* experiments demonstrated that intraperitoneal injection of BSN (75, 150 mg/kg) for 11 days significantly inhibited the growth of RKO xenografts and reduced GPX4 expression in tumour tissue (Wen et al., 2025). In summary, BSN induces ferroptosis by inhibiting p62/NRF2/HO-1 and downregulating GPX4, making it a potential candidate compound for the treatment of CRC. This study has limitations: only one ferroptosis inhibitor was used, and no Nrf2 agonist was employed for cross-validation.

Penexanthone A (PXA) is a flavonoid dimer isolated from the metabolites of the endophytic fungus Diaporthe goulteri, which is derived from *Candus ilicifolius* L. This study investigated human CRC using HCT116 and HT29 cell models, as well as zebrafish xenograft models, to elucidate the mechanism by which PXA enhances the chemotherapeutic sensitivity of cisplatin (CDDP). *In vitro* results showed that PXA (4–8 μM) synergistically inhibited CRC cell proliferation with cisplatin, activated the endogenous mitochondrial apoptosis pathway, significantly increased ROS levels, exacerbated DNA damage, and downregulated antioxidant systems such as GSH, SOD and HO-1, resulting in severe OS. PXA (0.1, 10 μg/mL) specifically inhibited Nrf2, blocked its downstream SLC7A11/GPX4 axis, thereby inducing ferroptosis. Overexpression of Nrf2 reversed these effects. *In vivo* experiments indicated that the combination of PXA and cisplatin significantly reduced the fluorescence intensity and proliferation area of xenograft tumours in zebrafish, enhancing the antitumour effect of cisplatin (Zhao et al., 2024). However, research is currently limited to the cellular and zebrafish levels, and further validation in mammals, as well as studies on long-term toxicity, pharmacokinetics and clinical translation are still required.

S-allylmercaptocysteine (SAMC) is an organosulphur compound derived from *Candus ilicifolius* L. that possesses anti-inflammatory and antitumour properties. Li et al. combined SAMC with the mTOR inhibitor rapamycin to systematically investigate its anti-CRC effects and molecular mechanisms in HCT-116 CRC cells and nude mouse xenograft models. *In vitro* results demonstrated that both drugs when used alone could inhibit cell proliferation. When combined, they synergistically enhanced this inhibitory effect and triggered characteristic apoptotic morphological changes. *In vivo* experiments confirmed that the combination of rapamycin and SAMC (300 mg/kg) significantly enhanced tumour suppression whilst mitigating rapamycin-induced weight loss, demonstrating superior safety. The combination induced apoptosis through the endogenous mitochondrial apoptotic pathway, accompanied by an elevated Bax/Bcl-2 ratio and upregulated p53 expression. Increased LC3-II further activated autophagy. It also abrogated rapamycin-mediated Akt phosphorylation to overcome drug resistance. Moreover, downregulation of p62 and activation of the Nrf2/NQO1 pathway boosted the activities of antioxidant enzymes including SOD, GSH-Px and CAT, and lowered MDA levels. Ultimately, the crosstalk among apoptosis, autophagy and the p62/Nrf2 axis mediated the synergistic antitumor action (Li et al., 2017). This study provides experimental evidence for the combined treatment of CRC with natural products and mTOR inhibitors. However, the optimal ratio, synergistic threshold, pharmacokinetics and long-term safety still require further investigation.

Research by Mai et al. revealed that the faecal metabolite citraconate can inhibit ferroptosis via the Nrf2 pathway, thereby driving the malignant progression of CRC. *In vitro* experiments showed that treatment of HCT116 cells with citraconate (12 mM) and MC38 cells with citraconate (9 mM) significantly enhanced cell proliferation, colony formation, migration and invasion capabilities. In in vivo mouse models, water-based citraconate (3 mM) intervention significantly increased the burden of liver metastases following splenic injection, without affecting body weight. Mechanistically, citraconate elevated Nrf2 protein levels, enhanced Nrf2 protein stability and nuclear translocation, promoted the transcriptional expression of downstream NQO1, GCLC and GCLM, and increased γ-glutamylpeptide synthesis, thereby reducing intracellular ferrous ion and MDA levels and inhibiting ferroptosis. Co-administration with the ferroptosis inducer RSL3 reversed its pro-tumour effects (Mai et al., 2024). This indicates that citraconate inhibits ferroptosis in an Nrf2-dependent manner, accelerating the progression of CRC. The study did not elucidate the specific mechanism by which it regulates Nrf2 degradation, nor was it validated in clinical cohorts. Therefore, the conclusions require further refinement through larger sample sizes and in-depth mechanistic studies.

### 5.9 Extracts

Brusatol is a chalcone compound extracted from the fruits of the plant *Coptis chinensis* Franch (family Simaroubaceae), possessing antitumour, anti-inflammatory and antioxidant activities. Liu et al. investigated its effects on CRC cells and the underlying mechanisms through experimental studies. *In vitro* experiments showed that Brusatol (15–240 nmol/L) significantly inhibited cell viability, live cell counts and clonogenic capacity, and suppressed the proliferation of HCT116 and HT29 cells. Brusatol (60 nmol/L) rapidly and transiently inhibited Nrf2 protein expression and, within 8 h, time-dependently suppressed the transcription of Nrf2 target genes G6PD, 6GPD, IDH1 and ME1, reduced cellular NADPH and GSH levels, and blocked DNA synthesis. Simultaneously, it decreased the G_0_/G_1_ and G_2_/M phase ratios, arrested cells in the S phase. The results indicate that Brusatol inhibits CRC cell proliferation by suppressing Nrf2, disrupting metabolic reprogramming and inducing S-phase arrest, without inducing significant apoptosis (Liu et al., 2018). This study is limited to *in vitro* validation and lacks *in vivo* and clinical data. The precise molecular mechanisms and the value of combination therapy require further investigation.

### 5.10 The role of traditional Chinese medicine formulations in the treatment of colorectal cancer

TCM formulas play a significant role in the treatment of CRC, exhibiting antitumor, anti-inflammatory, and immunomodulatory effects. They may exert their effects by regulating Nrf2-related pathways, inducing ferroptosis, inhibiting cell proliferation, migration, and invasion, or by enhancing anticancer effects in combination with Western medications to alleviate disease progression.

Shaoyao Decoction (SYD), derived from Liu Wansu’s “Su Wen Bing Ji Qi Yi Bao Ming Ji” of the Jin Dynasty, consists of nine Chinese medicinal herbs: *Areca catechu* L., *Rheum palmatum* L., *A. sinensis* (Oliv.) Diels, *Glycyrrhiza uralensis* Fisch., *Paeonia lactiflora* Pall., *Dolomiaea costus* (Falc.) Kasana and A.K.Pandey, *Neolitsea cassia* (L.) Kosterm., *C. chinensis* Franch., *Scutellaria baicalensis* Georgi. It is a classic formula for treating diarrhoea and dysentery caused by damp-heat, and holds potential for the prevention and treatment of ulcerative colitis and CRC associated with colitis. Wang et al. utilised AOM/DSS-induced mouse models and H_2_O_2_-induced HT-29 cell models to investigate its mechanism of action. *In vivo*, treatment with SYD (18.5 g/kg) significantly improved weight loss, colon shortening and tumour proliferation in mice. *In vitro*, SYD (0–8 mg/mL) alleviated oxidative damage and enhanced cell viability. Mechanistically, SYD promoted Nrf2 nuclear translocation and upregulated Nrf2 downstream antioxidant enzymes such as HO-1, NQO-1, GSTM1, GR, TR and other Nrf2 downstream antioxidant enzymes, whilst increasing SOD activity. Simultaneously, it inhibited Keap1 expression, reduced excessive ROS accumulation and elevated MDA levels. Furthermore, SYD inhibited NF-κB activation and reduced inflammatory factors such as TNF-α, IL-1β and ICAM-1, exerting antioxidant, anti-inflammatory and anti-proliferative effects, thereby blocking the progression of colitis to CRC (Wang et al., 2020). This study provides experimental evidence for the application of SYD. However, the models employed were limited to single-cell and animal studies, and the core active components of SYD were not identified. Furthermore, no dose-response or clinical translation studies were conducted.

Qingjie Fuzheng Granules is a TCM formula clinically used for the prevention and treatment of CRC, formulated with a balanced combination of *Scleromitrion diffusum* (Willd.) R.J.Wang, *Scutellaria barbata* D. Don, *A. mongholicus* Bunge, *Hordeum vulgare* L. In this study, using human CRC HCT-116 cells as models, it was found that following treatment with Qingjie Fuzheng Granules (0.5, 1.2 mg/mL), the drug significantly inhibited tumour cell proliferation, exhibiting a clear dose-dependent effect. Transmission electron microscopy revealed typical ultrastructural changes associated with ferroptosis, including mitochondrial atrophy, cristae fragmentation and even vacuolization. Simultaneously, it increased intracellular iron accumulation, ROS and the LPO product MDA, whilst decreased levels of the antioxidants GSH and SOD, indicating a significant disruption of redox homeostasis. Further mechanistic studies confirmed that Qingjie Fuzheng Granules significantly downregulated the expression of Nrf2, SLC7A11 and GPX4 proteins, thereby inhibiting Nrf2/SLC7A11/GPX4 to initiate the ferroptosis programme and exert an anti-CRC effect (Wu et al., 2026). This study elucidates the action pathways and targets at the *in vitro* cellular level, providing experimental evidence for the targeted treatment of CRC with TCM. However, the study has limitations: it utilised only a single cell line, lacks *in vivo* and clinical validation, did not conduct pathway rescue experiments, and has not yet identified the core active ingredients or the mechanism of the formula’s synergy.

Xihuang Capsules are a classic compound formula from the Qing Dynasty, composed of *Calculus* Bovis, *Moschus Artifactus*, *Boswellia sacra* Flück., and *Commiphora myrrha* (T.Nees) Engl. They possess anti-inflammatory, anti-tumour and immunomodulatory effects and are commonly used in the adjuvant treatment of malignant tumours. In their study, Liang et al. used CRC HCT-116 cells as *in vitro* models, employing 5.28% Xihuang Capsules and 170.20 mg/L cetuximab. The results showed that both drugs, when used alone, could inhibit cell proliferation, promote apoptosis, and reduce migration and invasion capabilities. The combined effect was even more pronounced. Mechanistically, the combination therapy inhibited the expression and pathway activation of Nrf2 and HO-1, suppressed MMP2, MMP9, ZNF320, VEGFA and GSK3β, whilst inhibiting BCL-2 and reducing the BCL-2/BAX ratio. This reduced the cells’ antioxidant capacity and drug resistance, enhanced tumour cell sensitivity to the drugs, and synergistically promoted apoptosis, suggesting that the combination exerts a synergistic anti-tumour effect by inhibiting the Nrf2/HO-1 pathway (Liang et al., 2024). This study is limited to *in vitro* experiments and lacks *in vivo* and clinical evidence. It has not identified the core active monomer or the complete regulatory network, resulting in limited clinical translation. Future work should include *in vivo* experiments, the isolation of active components, and the validation of targets to provide more robust evidence for the use of this combination regimen in the treatment of CRC.

The Fushaoshidiqin Formula (FSDQ) is an empirical formula developed for the treatment of CRC based on the theory of the pathogenesis of cancerous toxins and the ‘warming, nourishing and detoxifying’ method proposed by Master of TCM Zhou Zhongying. It is composed of six herbs: *P. lactiflora* Pall., *G. uralensis* Fisch., *Aconitum carmichaelii* Debeaux, *S. baicalensis* Georgi, *Sophora flavescens* Aiton, *Rehmannia glutinosa* (Gaertn.) Libosch. ex DC. *In vitro*, using CRC CT-26 cells as models, treatment with FSDQ (0.25, 0.5, 1 mg/mL) inhibited cell proliferation and migration, promoted increases in intracellular Fe^2+^, ROS and MDA levels, and upregulated Keap1 protein expression, whilst inhibiting Nrf2 nuclear transcriptional activity and the expression of SLC7A11 and GPX4, thereby inducing ferroptosis. *In vivo*, using oxaliplatin (1.5 mg/kg/day) as a positive control, continuous oral administration of FSDQ (4.49, 8.97, 17.94 g/kg) for 21 days significantly reduced tumour volume, increased Fe^2+^, ROS and MDA levels in mouse xenografts, and enhanced tumour tissue necrosis (Zheng et al., 2024). The results indicate that FSDQ induces ferroptosis in CRC cells by inhibiting Nrf2/SLC7A11/GPX4. This study has limitations, including a single model, a lack of clinical and pathway-reverse validation, and the failure to identify core active components. Further work is required to refine *in vivo* validation, confirm targets, and screen for components, thereby providing more robust evidence for clinical translation.

Zuo Jin Pill (ZJP) is a classic TCM formula derived from “Danxi Xinfang”, composed of *C. chinensis* Franch. And *Tetradium ruticarpum* (A.Juss.) T.G.Hartley in a 6:1 ratio. It possesses anti-tumour and drug resistance-reversing potential. However, the mechanism by which it reverses resistance to cetuximab (CET) in KRAS-mutated CRC remains unclear. This study utilised three KRAS-mutant CRC cell lines include HCT116, Lovo and SW620 as models to investigate the effects and mechanisms of ZJP in combination with CET. The experiment employed combined treatment with ZJP (50 μg/mL) and CET (125 μg/mL). The results showed that monotherapy had a weak inhibitory effect on cell proliferation, whereas the combination significantly reduced cell viability. Mechanistically, ZJP promoted the massive accumulation of intracellular ROS, increased Fe^2+^ levels and decreased GSH levels. These effects are further enhanced when combined with CET. Simultaneously, the expression of SLC7A11, GPX4, Nrf2 and MDM2 was decreased at both protein and mRNA levels. Nrf2-driven antioxidant defense was weakened, while p53 protein was stabilized and upregulated. These changes triggered ferroptosis and resulted in the death of drug-resistant cells. Furthermore, ferroptosis inhibitors were able to block this biological effect (Wei et al., 2023). The study confirmed that ZJP restores the sensitivity of KRAS-mutant cells to CET by regulating Nrf2/p53 and inducing ferroptosis via inhibition of the SLC7A11/GPX4 axis. As this study is limited to *in vitro* experiments and lacks *in vivo* validation and analysis of active components, further research is required to elucidate the mechanisms and pharmacodynamic effects, thereby providing a basis for clinically reversing targeted drug resistance.

## 6 Discussion

The Nrf2 signalling axis, as a central molecular hub regulating cellular redox homeostasis, inflammatory responses and cell fate determination, plays a “double-edged sword” role in the development, invasion, metastasis and treatment resistance of CRC. Under physiological conditions, Nrf2 exerts a protective effect on normal tissues by activating antioxidant target genes to maintain the integrity of the intestinal mucosal barrier, scavenge ROS, and suppress excessive chronic inflammation. However, in the CRC microenvironment, Nrf2 is frequently overexpressed and translocates to the nucleus due to Keap1 mutations, abnormal activation of upstream kinases, and impaired ubiquitination-mediated degradation. By enhancing tumour cells’ antioxidant capacity, inhibiting ferroptosis and apoptosis, driving abnormal cell cycle progression, promoting EMT, and reshaping the immunosuppressive microenvironment, it ultimately drives malignant proliferation, distant metastasis, and resistance to chemotherapy and targeted therapies. Consequently, the precise and selective regulation of the Nrf2 signalling axis rather than its simple activation or inhibition has emerged as a strategy of significant translational value in the prevention and treatment of CRC.

Natural products with their unique advantages of widespread availability, structural diversity, mild toxicity and side effects, and multi-component synergistic multi-target action, have become ideal candidate resources for targeting the Nrf2 pathway. This paper comprehensively summarises various naturally active substances, including flavonoids, terpenoids, alkaloids, glycosides, phenolics, coumarins, polysaccharides and TCM formulations. By inhibiting Nrf2 nuclear translocation, downregulating the expression of key proteins in the pathway, blocking downstream antioxidant and anti-apoptotic axes, and repairing OS-induced cellular damage, they achieve multiple effects including inhibiting the proliferation of CRC cells, inducing programmed cell death, reversing chemotherapy resistance, and alleviating chemotherapy-related intestinal mucosal damage. This provides a solid experimental basis and theoretical support for the development of safe and effective interventions against CRC. From the perspective of mechanisms of action, the regulation of Nrf2 by natural products exhibits a high degree of context-dependence. In tumour cells, most natural monomers such as quercetin, genistein, coptisine and stephanin primarily act to inhibit the Nrf2 pathway. They suppress invasion and metastasis by disrupting the tumour cells’ antioxidant defence system, exacerbating ROS accumulation, triggering ferroptosis and mitochondrial apoptosis, and blocking the EMT process. Conversely, in normal intestinal epithelium or chemotherapy-induced injury models, certain natural products such as iridoflavone, astragaloside A, procyanidin B2 and Paeoniae Decoction can moderately activate Nrf2, thereby enhancing tissue antioxidant and anti-inflammatory capacity, protecting the mucosal barrier and reducing abnormal cell apoptosis. This bidirectional regulatory property enables natural products to achieve antitumour effects whilst reducing toxicity to normal tissues, thereby addressing the shortcomings of traditional chemotherapeutic agents, namely, their poor selectivity and severe adverse reactions. Furthermore, extensive research has confirmed that the combined use of natural products with chemotherapeutic agents can significantly enhance anticancer efficacy and reverse drug resistance through synergistic regulation of the Nrf2 pathway. Examples include the combination of quercetin with 5-fluorouracil, DMY combined with oxaliplatin, and ZJW combined with cetuximab, all provide new approaches for overcoming drug resistance in clinical settings by inhibiting Nrf2-downstream resistance-associated proteins, enhancing intracellular drug accumulation, and intensifying OS and cell death.

Although natural products targeting Nrf2 for the treatment of CRC have demonstrated significant potential, current research remains at the preclinical stage, with numerous bottlenecks in mechanism elucidation, model construction, drug development and translational applications. Firstly, most studies have remained at the level of macroscopic detection of Nrf2 protein expression and nuclear translocation, lacking structure-activity evidence of direct binding between natural products and key molecules such as Nrf2 and Keap1. The analysis of upstream regulatory kinases, ubiquitination modifications, non-canonical degradation pathways, and cross-signalling networks has not been sufficiently systematic. In particular, the mechanisms by which multi-component, multi-target TCM formulations synergistically regulate Nrf2 remain unclear. Secondly, existing research relies heavily on *in vitro* cell lines and xenograft tumour models, which struggle to simulate the tumour heterogeneity, immune microenvironment, and interactions with the gut microbiota found in human CRC. There is a lack of validation using organoids, genetically engineered mice, and clinical samples, resulting in limited generalisability of the findings. Thirdly, natural products generally suffer from poor water solubility, low oral bioavailability, significant first-pass metabolism in the liver, and insufficient targeted accumulation *in vivo*. Most studies have not conducted systematic evaluations of pharmacokinetics, dose-response relationships, or long-term safety, making it difficult to support clinical translation. Fourthly, the physiological protective function of Nrf2 in normal tissues cannot be overlooked. Non-selective inhibition may increase the risk of oxidative damage and inflammation. However, there is currently a lack of precise regulatory strategies capable of distinguishing between tumour and normal tissues, and the safety margins and therapeutic window remain unclear. Finally, although TCM formulations demonstrate outstanding overall efficacy, the absence of studies on the separation of individual components, the identification of core active substances, and the elucidation of combination mechanisms makes it difficult to meet the standardisation and quality control requirements of modern drug development.

Looking ahead, research into natural product-based Nrf2-targeted therapies for CRC must advance in a more precise, systematic and translational direction. At the mechanistic level, techniques such as molecular docking, SPR, CRISPR-Cas9 and single-cell sequencing should be utilised to identify the direct binding sites of natural bioactive compounds with Nrf2, construct an interactive regulatory network between Nrf2 and pathways such as ferroptosis, apoptosis, autophagy, NF-κB, STAT3 and Wnt/β-catenin, and systematically elucidate the scientific rationale behind the formulation of TCM compounds. At the model level, research involving patient-derived organoids, immunocompetent mice and clinical cohort studies should be advanced to improve the alignment of experimental results with real-world clinical scenarios. At the drug development level, strategies such as structural modification, nanodelivery and gut-targeted formulations should be employed to enhance bioavailability and tumour accumulation, whilst establishing standardised systems for evaluating efficacy and safety. At the clinical translation stage, precision intervention protocols should be developed based on Nrf2 expression levels, Keap1 mutation status and tumour molecular subtypes. Optimal combination regimens of natural products with chemotherapy, targeted therapy and immunotherapy should be explored, and a system of efficacy monitoring biomarkers centred on Nrf2, HO-1, GPX4 and SLC7A11 should be established.

In summary, natural products offer an innovative pathway for the prevention and treatment of CRC through the precise regulation of the Nrf2 signalling axis. Their advantages of multi-targeted action, low toxicity and bidirectional regulation align with the development trends of modern precision and adjuvant cancer therapies. Several challenges still exist, including unclear molecular mechanisms, inadequate translational research and non-ideal formulation specifications. With the development of interdisciplinary disciplines and technological innovations, naturally occurring Nrf2 modulators are promising adjunctive agents for CRC prevention and treatment. They can improve clinical outcomes, ameliorate patient prognosis and mitigate treatment-induced toxicity.

## Glossary

CRC: Colorectal cancer
TCM: traditional Chinese medicine
Nrf2: Nuclear Factor Erythroid 2-Related Factor 2
OS: oxidative stress
EMT: epithelial-mesenchymal transition
bZIP: basic zinc-finger
ROS: Reactive Oxygen Species
ARE: antioxidant response element
GPX4: Glutathione Peroxidase 4
SOD: superoxide dismutase
GPx: glutathione peroxidase
HO-1: haem oxygenase-1
NO: nitric oxide
NQO1: NAD(P)H: Quinone Oxidoreductase 1
LPO: Lipid Peroxidation
TEC: Tectorigenin
TAX: Taxifolin
CyCl: Cyanidin chloride
FN: Formononetin
DMY: Dihydromyricetin
TC: Tagitinin C
TPL: Triptolide
AS-IV: Astragaloside IV
PL: Piperlongumine
Cep: Cepharanthine
ALO: Aloperine
QC: quinacrine
EGCG: Epigallocatechin gallate
HT: hydroxytyrosol
PB2: procyanidin B2
CMP: Carboxymethyl Pachyman
Are: Arenobufagin
BSN: Brassinin
PXA: Penexanthone A
SAMC: S-allylmercaptocysteine
SYD: Shaoyao Decoction
FSDQ: Fushaoshidiqin Formula
